# Chromosome-Level Genome Sequences, Comparative Genomic Analyses, and Secondary-Metabolite Biosynthesis Evaluation of the Medicinal Edible Mushroom Laetiporus sulphureus

**DOI:** 10.1128/spectrum.02439-22

**Published:** 2022-10-06

**Authors:** Wei-ge Dong, Zhen-xin Wang, Xi-long Feng, Rui-qi Zhang, Dao-yin Shen, Shuangtian Du, Jin-ming Gao, Jianzhao Qi

**Affiliations:** a Shaanxi Key Laboratory of Natural Products & Chemical Biology, College of Chemistry & Pharmacy, Northwest A&F University, Yangling, Shaanxi, China; b Yangling Zhijun Fungi Biotechnology Engineering Co., Ltd., Yangling, Shaanxi, China; The Ohio State University

**Keywords:** *Laetiporus sulphureus*, pharmaceutical edible mushroom, near-chromosome level assembly, secondary metabolite, biosynthetic potential

## Abstract

Laetiporus sulphureus mushroom is a complementary and alternative medicine that has anticancer, antioxidation, and analgesic effects and immunomodulatory activity; it is used as a treatment for cough and rheumatism and is a functional food that can improve physical fitness. Even though L. sulphureus has garnered considerable biotechnological and pharmacological interest due to its excellent cellulose-degrading ability and diverse biological activities, its biosynthetic potential regarding polysaccharides and secondary metabolites has not been thoroughly examined. In this study, we sequenced and assembled the whole genome of a wild *L. sulphureus* isolate, NWAFU-1, from the Qinling Mountains in China. Comparative genomes analysis revealed genomic differences between subspecies, and phylogenomic analysis revealed evolutionary divergence as well as genome expansion and contraction of individual Polyporaceae family species. Bioinformatics investigation identified candidate genes associated with mating type, polysaccharide synthesis, carbohydrate-active enzymes, and secondary-metabolite biosynthesis, which included multiple terpenoids, nonribosomal peptides, and polyketides. The locations of biosynthetic core genes were mapped and displayed on chromosomes and contigs. Totals of 143 proteins from 126 coding genes were identified and divided into 14 cytochrome P450 families. Furthermore, the biosynthetic network of tetracyclic triterpenoid active components was postulated by genome mining of related genes combined with the molecular network of metabolites. The genome analysis of *L. sulphureus* in this study improves the understanding of the biosynthesis of active compounds, which will lay a theoretical foundation for subsequent research on active-compound biosynthesis and promote the application of *Laetiporus* in the field of drug research and functional-food creation.

**IMPORTANCE** *L. sulphureus* is a parasitic basidiomycete fungus that causes brown rot. The fruiting bodies of *L. sulphureus* are used as ancient medicines in China and Europe to cure cancer, analgesia, cough, and rheumatism and are considered a functional food that regulates the body and improves health. *L. sulphureus* was inferred to be a tetrapolar system based on a high-quality genome, which will aid molecular breeding and artificial farming. Screening polysaccharide synthesis candidate genes and comparing carbohydrate-associated genes in brown-rot basidiomycetes help understand their growth. Identifying core genes for secondary-metabolite biosynthesis, gene cluster family analysis, and comparative cluster analysis will guide heterologous-biosynthesis investigations of these genes and help elucidate the biosynthetic pathways for *L. sulphureus* bioactive natural components. The biosynthesis network of tetracyclic triterpenes was mapped using metabolite profiling and genome scanning. This work explores the biosynthetic capacity of *L. sulphureus*-derived natural products and lays the foundation for biosynthetic studies of them.

## INTRODUCTION

Higher fungi, mainly belonging to the phylum Basidiomycota, are commonly called mushrooms due to their ability to form fruiting bodies. Mushrooms have been extensively used in human life for thousands of years due to their unique edible and/or medicinal properties (1, 2). Mushrooms are one of the healthiest food groups in the world, and approximately 50% of edible mushrooms are considered functional foods, making them recognized as superfoods (3). The genus *Laetiporus* Murrill was first established by Murrill in 1904 (4), and the genus belongs to the order Polyporaceae of the class Agaricomycetes (Basidiomycota) (5). A multigene phylogenetic analysis revealed at least 17 species of the genus *Laetiporus* (6). The fungi of the genus *Laetiporus* Murrill are both highly damaging forest pathogens, parasitic on 20 species of living trees worldwide (7), including *Quercus* and *Eucalyptus* (8), and brown-rot type wood decay basidiomycetes (9), which are essential to nature’s carbon cycle due to their highly specialized biomass degradation capacity (10).

Laetiporus sulphureus (Bull.) Murrill is a model strain of the genus *Laetiporus* (4), which has received figurative and trivial names such as “chicken mushroom” or “wood chicken” for the shape, size, and color of its fruiting bodies (11). As wood-inhabiting macrofungi with high medicinal and edible value, the fruiting bodies of *Laetiporus sulphureus* have a long tradition of consumption in Europe, America, and China (12), and their medicinal uses are documented in the ancient books of traditional Chinese medicine. The bioactive compounds and extracts of *L. sulphureus* and their benefits for human health have been thoroughly investigated in recent years. The triterpenoids, represented by eburicoic acid, are well-known bioactive metabolites from *L. sulphureus*, with significant anticancer, cytotoxic, and anti-immune activities (11, 13). Several sesquiterpenoids from *L. sulphureus* also exhibit anticancer activity (14, 15). Antibacterial and antioxidative properties are one of the distinguishing features of *Laetiporus sulphureus* metabolites (16–19). Laetiporic acids derived from *L. sulphureus* exhibit remarkable antifungal activity against the protoplasts of Aspergillus (20) and are a group of nonisoprene polyenes containing multiple conjugated double bonds and natural dyes with food colorant potential (21, 22). In addition, the crude polysaccharides from *Laetiporus sulphureus* exhibited antidiabetic effects by increasing the proliferation and insulin secretion function of rat insulinoma RINm5F cells (23). In addition, the fruit body of *L. sulphureus* can be used as an inexpensive and safe biomass due to its high content of α-(1→3)-glucans, which have been artificially cultivated (24).

The rapid advances in genome sequencing technologies have led to investigations of the medicinal active ingredients of wood-dwelling macrofungi and their biosynthesis increasingly relying on genome sequences rather than just on the metabolites themselves. The resolution of genomic information on wood-dwelling macrofungi will help to understand their life cycle, mating types, and nutritional models. To date, the genomes of several valuable medicinal (edible) fungi, including Ganoderma lucidum (25, 26), Inonotus obliquus (27), Hericium erinaceus (28), Naematelia aurantialba (29), Antrodia camphorata (30), and Sanghuangporus sanghuang (31), have been deciphered, thereby further facilitating their medicinal utilization and industrial development. *L. sulphureus* is a wood-dwelling-type medical and edible fungus that has attracted much attention, although the genomes of its five subspecies (10, 32–34) have been sequenced and used to analyze the lignocellulosic biomass degradation characteristics (10, 33). However, genome mining for secondary-metabolite biosynthesis is rarely reported. This work presents the near-chromosomal-level genome assembly of a wild *L. sulphureus* strain from the Qinling Mountains (a famous mountain system in China). Comparative genomes reflect genomic differences among subspecies, and phylogenomic analysis reveals the timing of evolutionary divergence, genome expansion, and contraction of *L. sulphureus* and well-known species of Basidiomycota. Functional genes related to mating type, polysaccharide synthesis, and carbohydrate-active enzymes (CAZymes) were subjected to *in silico* analysis. Genome mining combined with metabolite profiling identified gene clusters involved in secondary-metabolite biosynthesis. A family classification analysis of cytochrome P450 enzymes associated with primary metabolism and postsecondary metabolic modifications was performed. The biosynthesis of tetracyclic triterpenes was systematically speculated based on the molecular network of metabolites. This investigation sheds light on the biosynthesis of active ingredients derived from *L. sulphureus*, which, in turn, supports their synthetic biology production and in-depth pharmacological studies.

## RESULTS

### Fungal strain, strain culture, and species identity.

**FIG 1:**
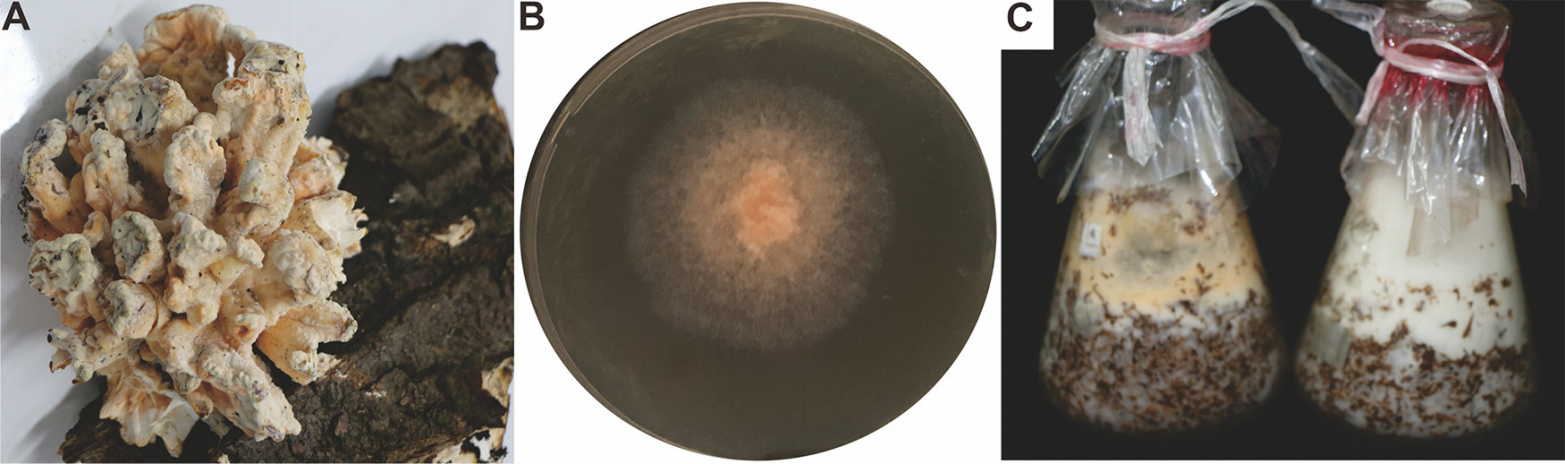
Morphologic photographs of strain *L. sulphureus* NWAFU-1: wild fruiting bodies (A), mycelium growing on PDA for 3 days (B), and mycelium growing on wood chips (left) and wheat bran (right) (C).

Wild fruiting bodies (Fig. 1A) were collected from the Qinling Mountains in Ankang City, Shaanxi Province, China, and were parasitic on the trunk of a living Toxicodendron vernicifluum (Stokes) F. A. Barkl tree. The culturable mycelium (Fig. 1B) was obtained by tissue separation of the fruiting body, which was able to grow vigorously on wheat bran and wood chips (Fig. 1C). Internal transcribed spacer (ITS) sequence alignment showed that the sample was 99.83% identical to *L. sulphureus* var. *miniatus* strain NAAS04758 (see Fig. S1 in the supplemental material). This sample was identified as *L. sulphureus* by combining ITS analysis and morphological characteristics of the fruiting bodies; the sample was named *L. sulphureus* NWAFU-1. Considering its rare food and medicine value and strain resources, *L. sulphureus* NWAFU-1 was subjected to genome sequencing.

### Genome sequence assembly and annotation.

**FIG 2:**
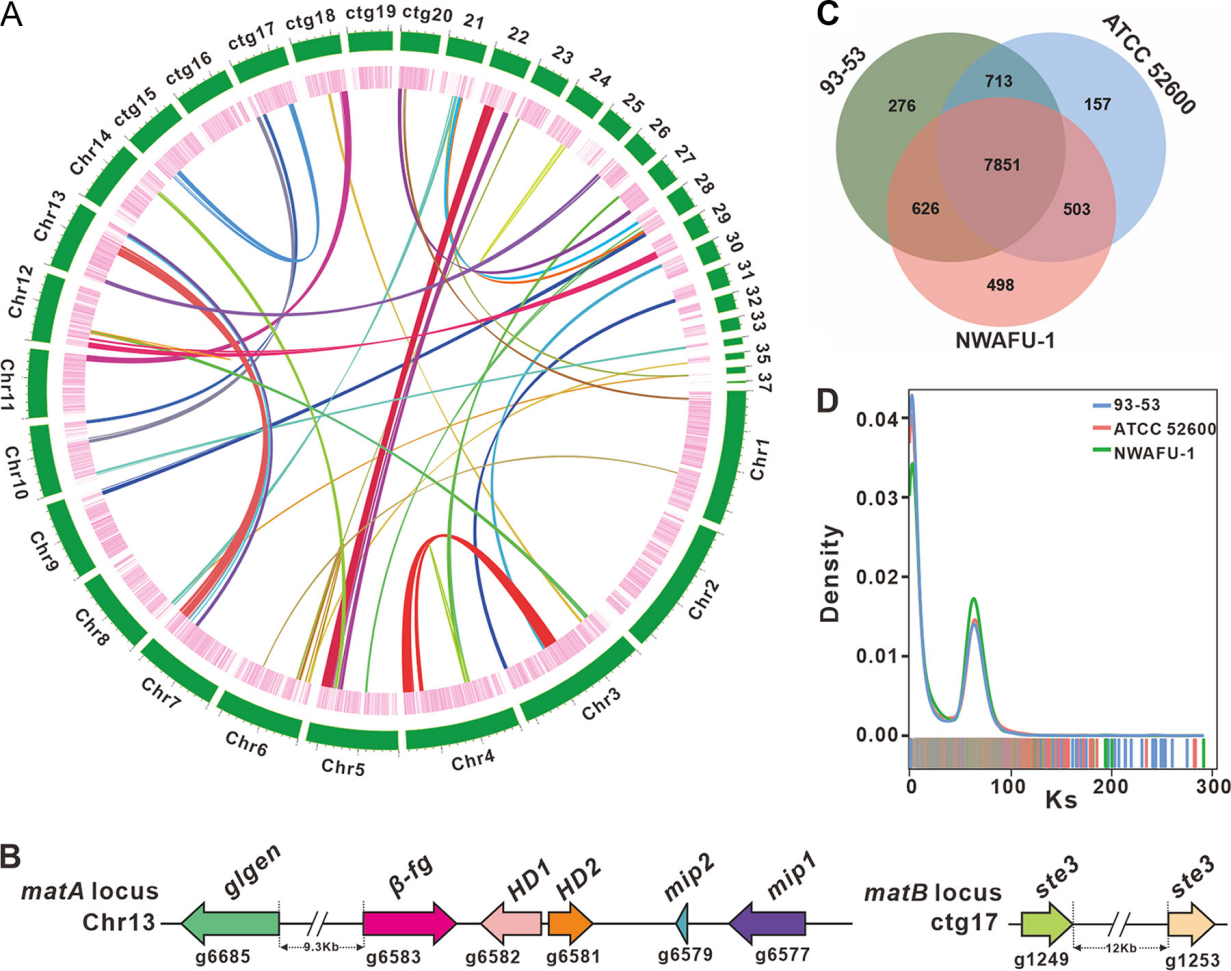
Genomic characterization, mating-type loci, and comparative genomic analysis. (A) Characteristics of the *de novo* assembly genomic features of *L. sulphureus* NWAFU-1. From the outside to the inside are chromosome and contigs, gene density (the intensity of the color positively correlates with gene density), and whole-genome collinearity analysis based on protein-coding genes (sequence similarity from low to high is indicated by yellow to red). (B) Structural diagram of the genes on the *matA* locus and *matB* locus. (C) Venn schematic of comparative genomes within *L. sulphureus* strains. (D) *K_s_* comparison within *L. sulphureus* species.

Totals of 11, 943, 393, 600 bases from 79, 622, 624 clean reads were assembled into the 48.23-Mb genome (Fig. 2A), which consisted of 14 chromosomal pseudomolecules and 23 contigs with an *N*_50_ of 1,791,120 bp and a 51.68% GC content (Tables S1 and S2). A k-mer analysis of reads showed two main peaks with similar heights, and the abscissa value of the former was half of the latter (Fig. S2). This result indicated that strain NWAFU-1 was a highly heterozygous species. Although the 99.81% coverage (Table S3) partly indicated that the genome of strain NWAFU-1 was well assembled, the BUSCO value was only 93.3% (Table S4). Sequencing, assembly parameters, and quality metrics comparisons between NWAFU-1 and reported *L. sulphureus* genomes further highlight the high-quality genome of strain NWAFU-1 (Table 1).

**TABLE 1:** Comparison of sequencing and assembly metrics, and genome quality of *L. sulphureus*

Parameter	NWAFU-1	93-53	ATCC 52600	Murrill, 1920	MG138
Sequencing technology	Illumina NovaSeq 6000	Illumina HiSeq 4000	Illumina HiSeq 2500	PacBio Sequel II HiFi Illumina NovaSeq 6000	Illumina HiSeq 4000
Sequencing depth	214.04×	85.2×	125.0×	106×	90×
No. of scaffolds	37	399	785	31	11,961
Total assembly length (bp)	48,323,753	39,909,705	43,372,605	37,410,216	51,778,905
Largest length (bp)	3,376,063	2,261,102	1,372,164	4,563,458	151,512
Scaffold *N*_50_ (bp)	1,791,120	522,580	211,056	2,582,225	14,997
Scaffold *L*_50_	10	22	53	6	862
GC content (%)	51.68	51.4	51.22	51.7	NA^*a*^
BUSCO completeness (%)	93.3	NA	NA	95.9	85.90
No. of predicted genes	15,302	13,774	12,802	NA	22,479
GenBank accession no.	GCA_024321985.1	GCA_001632365	GCA_016068325	GCA_927399515	GCA_003521245
Isolate information	Mycelium	NA	Mycelium	Mycelium	Mycelium
Reference	This study	33	10	34	32

a NA, not available.

There were 15,302 protein-coding genes predicted, with an average gene length of 1,272.37 bp and a total of 111,297 exons (average length, 174.94 bp) and 95,995 introns (average length, 74.68 bp) in these coding genes (Table S5). For noncoding RNA (ncRNA), 81 tRNAs, 50 rRNAs, 19 snRNAs, and 1 sRNA were predicted (Table S6). A total of 16,663 repeats with a total length of 9,067,448 bp was predicted, accounting for 18.76% of the entire genome, with the four scattered repeats short interspersed nuclear elements (SINE), long interspersed nuclear elements (LINE), long terminal repeats (LTR), and DNA transposons accounting for 0.00% (4), 0.26% (257), 4.93% (1,835), and 1.51% (1,024), respectively (Table S7).

To archive comprehensive protein-coding genes function annotation, 15,302 genes were subjected to sequence similarity analysis and motif similarity search based on nine public databases (Nr, Pfam, eggCOG, UniProt, KEGG, GO, Pathway, RefSeq, and Interproscan) (Table S8). The results of the Nr library annotation found that 94.14% of the 13,307 annotated genes matched the genome of *L. sulphureus* 93-53 (Fig. S3), indicating that NWAFU-1 is definitely of the *L. sulphureus* species. Cellular components constituted the main group among the 6,689 genes annotated by the functional classification of the GO database (Fig. S4). Functional annotation based on the COG database identified 1,350 genes, of which 154 belonged to the J group (translation, ribosomal structure, and biogenesis) (Fig. S5). According to the KEGG database, 5,010 genes were identified as being involved in 5 types of pathways, with the largest number of genes involved in metabolic pathways (Fig. S6). A domain-based motif search using the Pfam database identified 9,523 genes, and the top 20 types with the largest number are shown in Fig. S7. These various perspectives and levels of annotation demonstrate the functional diversity of protein-coding genes from strain NWAFU-1.

### Identification of the mating genes.

In the sexual development of mushroom-forming fungi, mating is an essential step. Genes associated with mating are in charge of directing the process. Mating-type (MAT) loci are located in distinct genomic regions that control mating (35). Heterozygous cooperation accounts for 90% of the sexual reproduction of basidiomycetes (36), which can be divided into bipolar and tetrapolar mating types. Among them, the tetrapolar mating-type system is by far the most extensive and complex sexual reproduction control system in Basidiomycetes, and its proportion is also the highest (37). Although *L. sulphureus* is a higher fungus of great interest, its mating type has not been identified.

In this study, the MAT-A locus was located on chromosome 14 (Chr13) by homology search with the mitochondrial intermediate peptidase (*mip*) codon gene and the glycosyltransferase (GT) family 8 protein codon gene (*glgen*) of four famous mushrooms, including Taiwanofungus camphoratus (Fig. S8A), and the MAT-B locus was located on contig 17 (ctg17) by scanning with the pheromone receptor genes (*ste3*) as a probe (Fig. S8B). The MAT-A locus contains two *mip* genes (g6577 and g6579), two homeodomain transcription factor codon genes (*HD1* and *HD2* [g6582 and g6581]), an unknown conserved fungal protein codon gene (*β-fg* [g6583]), and a *glgen* gene (g6685) about 9.3 kb from the *β-fg* gene. The MAT-B locus contains at least two unclustered *ste3* genes (g1249 and g1253) (Fig. 2B and Table S9). The analysis finding that the MAT-A locus and the MAT-B locus are not in the same contig suggests that the mating type of *L. sulphureus* has a tetrapolar mating system. Overall, further research is needed to better understand the genomic structure of the mating-type loci in *L. sulphureus*.

### Comparative genomic analysis within the *L. sulphureus* species.

The genome size of the strain NWAFU-1 is much larger than those of strains 95-53 and ATCC 52600, and accordingly, the number of protein genes encoded in NWAFU-1 is much larger than those in these two strains (Table 1). A total of 7,851 orthologous groups were identified from the three species of *Laetiporus*, and NWAFU-1 contained more unique orthologous groups (498) than did 93-53 (276) and ATCC 52600 (157). NWAFU-1 shared more orthologous groups with 93-53 (626) than ATCC 52600 (503), and the number of orthologous groups shared between the last two (713) was greater than the number they shared with NWAFU-1. This finding suggests that the similarity between 93-53 and ATCC 52600 is greater than the similarity of each of them to NWAFU-1 (Fig. 2C).

To further understand the differences in the genomes of the strains of *L. sulphureus*, a genome-wide duplication analysis based on synonymous mutation rates was performed. The consistent trends in the synonymous substitution *K_s_* curves of these strains revealed that they are all *L. sulphureus* species uniform (Fig. 2D). The obvious peaks in the *K_s_* curves suggested that genome doubling events occurred during the genomic evolution of the species *L. sulphureus* (Fig. 2D), as evidenced by genome-wide collinearity analysis of NWAFU-1 (Fig. 2A). The higher *K_s_* peaks of NWAFU-1 indicated that this strain has undergone a larger-scale genome doubling event (Fig. 2D), resulting in the larger genome of this strain (Table 1).

### Phylogenomic and evolutionary analysis of *L. sulphureus*.

**FIG 3:**
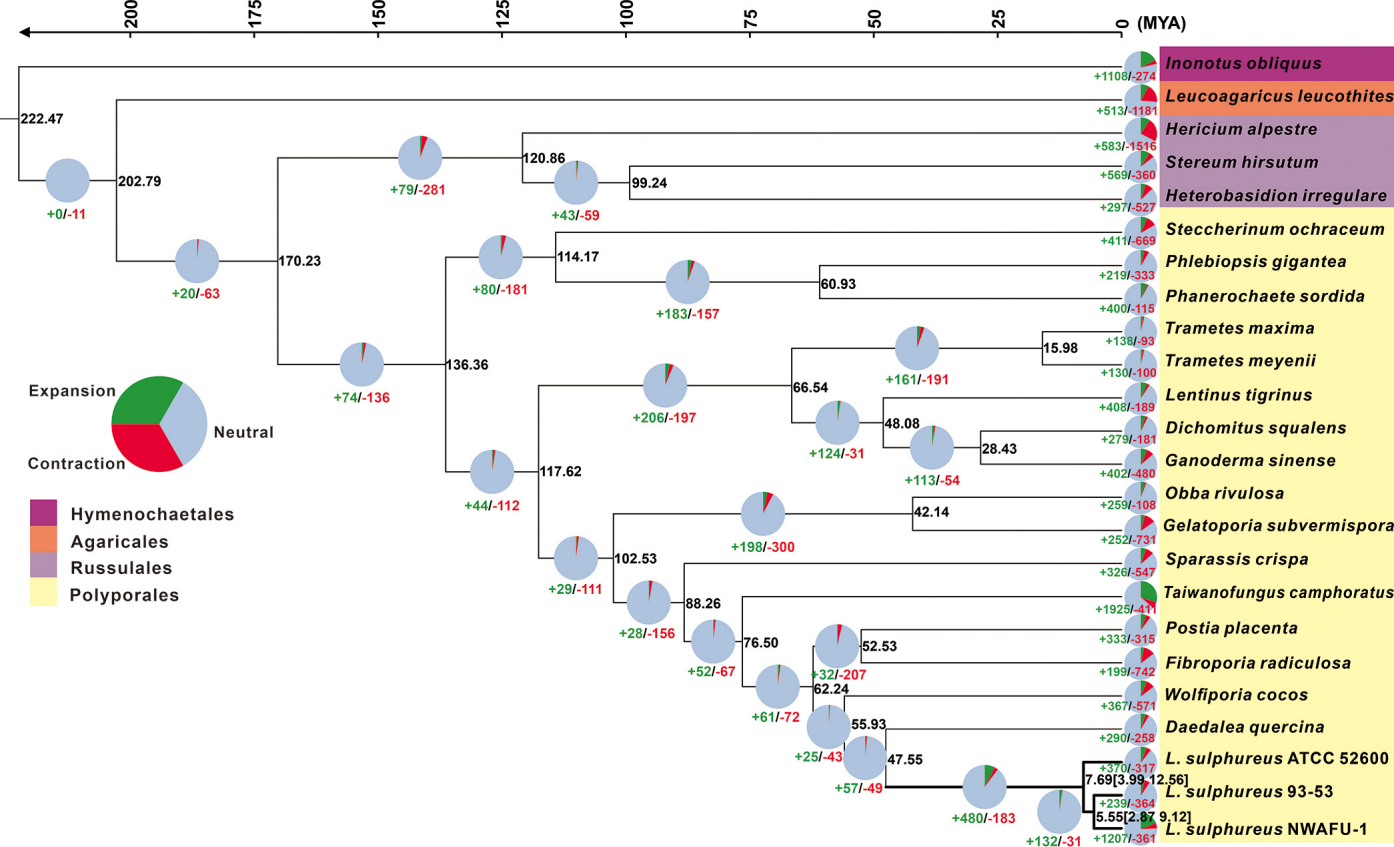
Maximum clade credibility tree inferred from 1,702 single-copy orthologous genes. All nodes received full bootstrap support. The divergence time is labeled as the mean crown age for each node, while the 95% highest posterior density is also given within the *Laetiporus* clade. The black numbers at the branches indicate the corresponding divergence times in MYA. The numbers of gene family expansion and contraction in each species are labeled with green and red symbols, respectively. The proportion of expansion, contraction, and neutrality in the genome of each species is displayed before the species name. The color of the background of each species indicates its corresponding order.

The phylogenomic tree inferred from an alignment of 579 single-copy orthologous genes from 264,508 proteins delimited phylogenetic relationships among 24 species, including three strains of *L. sulphureus*, with full bootstrap support (Fig. 3). The mean divergence time of the Hymenochaete outgroup represented by *Inonotus obliquus* is estimated to be 222.47 million years ago (MYA), while those of the Agaricales and Russulales are estimated to be 202.79 and 170.23 MYA, respectively. The divergence time between *L. sulphureus* and *Daedalea quercina* is estimated at 47.56 MYA. *L. sulphureus* was estimated to emerge in a mean crown age of 7.69 MYA, with a 95% highest posterior density (HPD) of 3.99 to 12.56 MYA, while the divergence time between subspecies 93-53 and NWAFU-1 was 5.55 MYA, with a 95% HPD of 2.87 to 9.12 MYA (Fig. 3). Besides three strains in *L. sulphureus*, other well-recognized medicinal fungal species from Polyporales, such as Ganoderma sinense, *T. camphoratus*, and Wolfiporia cocos, were also phylogenetically separated (Fig. 3).

The gene family contraction occurred more frequently than the gene family expansion in the evolutionary process of the 24 fungal species studied (Fig. 3). In the case of *L. sulphureus*, 370, 239, and 1,207 gene families had expanded in the strains ATCC 52600, 93-53, and NWAFU-1, corresponding to 317, 364, and 361 genes being contracted, respectively. Among these gene families, 122 (50 expanded and 72 constricted), 110 (40 expanded and 70 constricted), and 181 (119 expanded and 62 contracted) in ATCC 52600, 93-53, and NWAFU-1, respectively, have undergone significant evolution (Fig. 3).

### CAZyme analysis and synthesis of polysaccharides.

**FIG 4:**
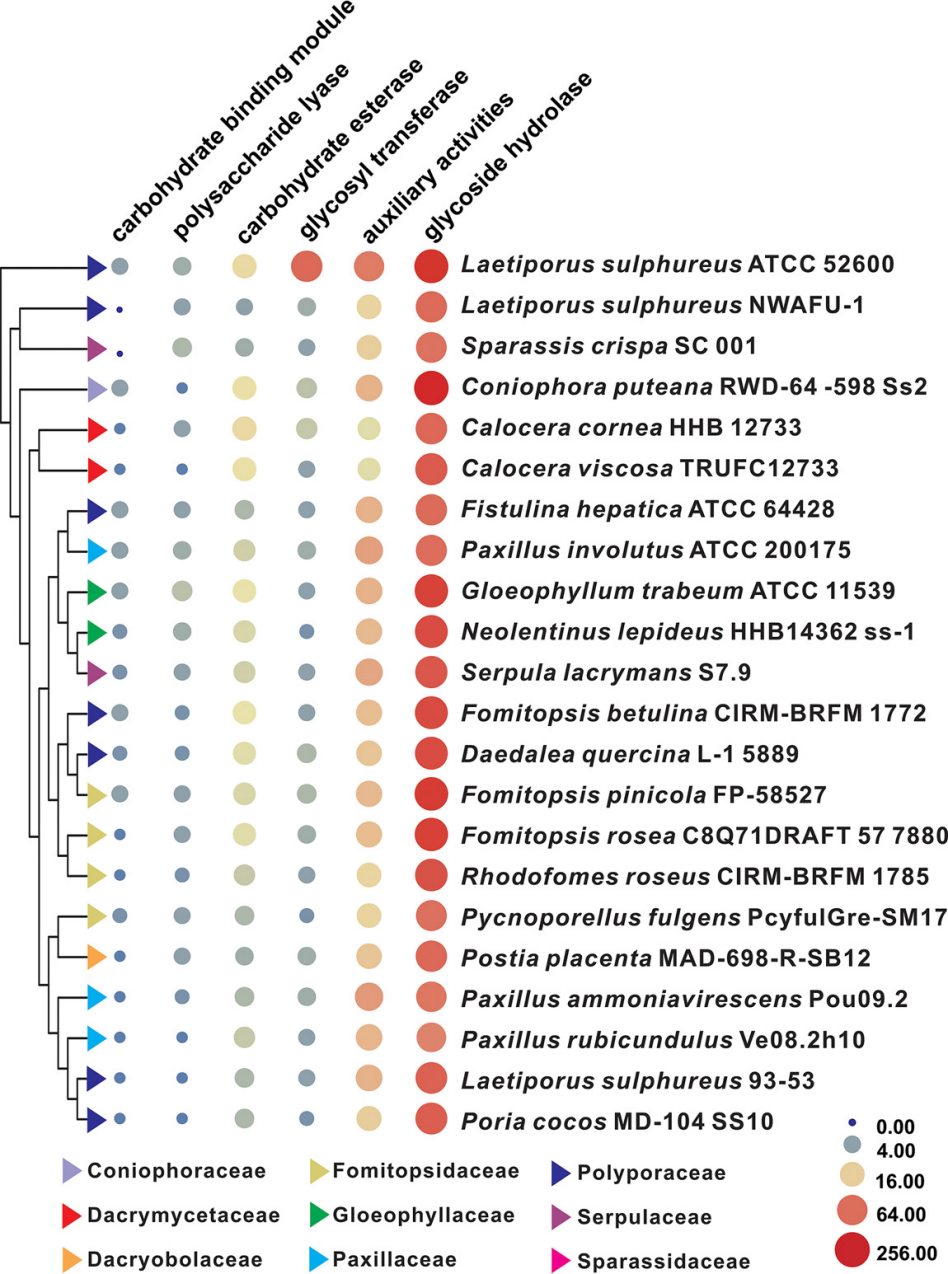
Bubble plot of CAZyme analysis of *L. sulphureus* and related brown-rot basidiomycetes. The sizes and colors (from blue through buff to red) of circles indicate the change in quantity, and the different colored triangles indicate different families.

In spite of the fact that the carbohydrate-active enzymes (CAZymes) of *L. sulphureus* ATCC 52600 have been identified using multiomics approaches (10), the differences in the genome piqued our interest in the CAZymes of NWAFU-1. There were 90 genes encoding CAZymes, including 66 glycoside hydrolases (GHs), 14 auxiliary activities (AAs), 4 GTs, 3 glycoesterases (CEs), and 3 polysaccharide lyases (PLs), but no carbohydrate-binding module (CBM) was found. The GH members g12742.t1 and g2925.t1 are of interest because they both contain two HG13 structural domains. Among these six classes of genes, the number of GH genes is much higher than those of the others, and the GHs encoded by most genes are mainly involved in the degradation of pectin (GH28), cellulose (GH5), chitin (GH18), hemicellulose (GH10 and GH43), and starch (GH13 and GH31) (Table S10). The CAZyme spectra of *L. sulphureus* and 19 typical brown-rot basidiomycetes were compared (Data Set S1), and it was discovered that the numbers and types of CAZymes in these strains were not species specific. The strain with the characteristics of CAZymes most similar to those NWAFU-1 was Sparassis crispa SC 001, rather than strain 93-53 or ATCC 52600 (Fig. 4).

Exopolysaccharides (EPS), intracellular polysaccharides (IPS), and other polysaccharides have been found in the fruiting bodies (38) and fermentation broth of mycelium (23, 39) of *L. sulphureus*. Approximately 20 different enzymes involved in mushroom polysaccharide synthesis have been identified (40–42), including 1,3-glucan synthase (GLS), glucose phosphomutase (PGM), phosphomannose isomerase (PMI), glucokinase (GK), beta-glucan synthesis-associated protein (GSAP), phosphoglucose isomerase (PGI), UDP-glucose 4-epimerase (UGE), GDP-mannose dehydratase (GMD), phosphofructokinase (FPK), and UDP-xylose synthase (UXS). With the aid of the genes involved in the polysaccharide synthesis pathway of Basidiomycetes, 23 polysaccharide biosynthetic candidate genes were identified, including 5 GSAPs, 4 GMDs, 3 UGEs, and 2 GKs, PGMs, and GLSs, as well as only 1 FPK, PGI, and UXS (Table S11). The number of genes involved in polysaccharide biosynthesis possessed by *L. sulphureus* is similar to that in other medicinal and edible mushrooms such as *H. erinaceus* (40).

### Secondary-metabolite biosynthetic gene cluster analysis.

**FIG 5:**
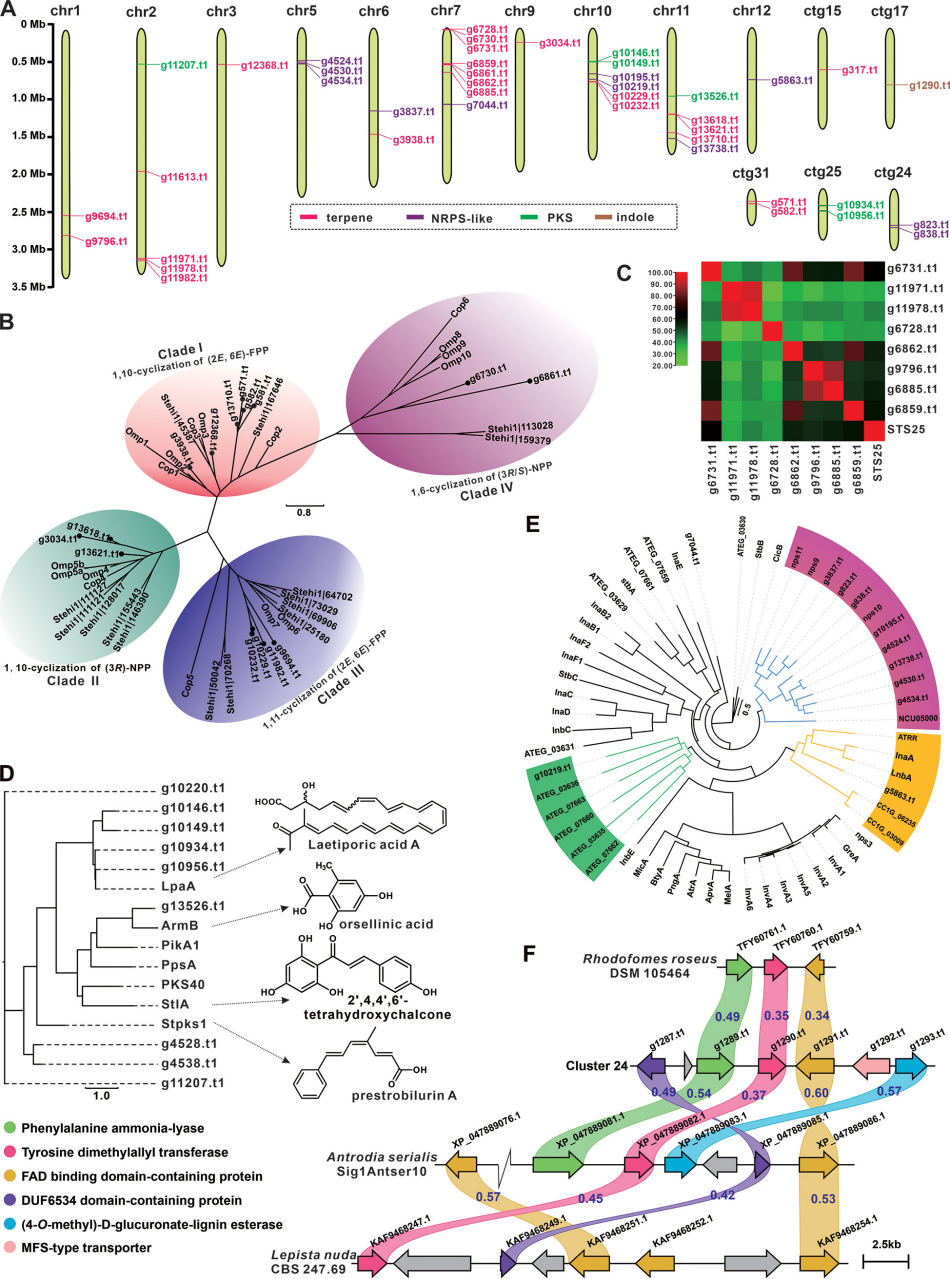
Analysis of genes involved in secondary-metabolite biosynthesis. (A) Distribution of biosynthetic core genes for natural products on the chromosomes (contigs). (B, D, and E) Phylogenetic tree analysis for sesquiterpene synthases (B), PKSs (D), and NRPS-like enzymes (E) from NWAFU-1 and their respective homologs. (C) Percent identity matrix of 8 STSs from NWAFU-1 and STS25. (F) Comparison of indole-type BGC and similar BGCs; similarity is marked between the corresponding two genes.

In view of the long medicinal history and significant medicinal value of *L. sulphureus*, we analyzed the biosynthetic potential of its secondary metabolites. The genome of NWAFU-1 was predicted using the webtool antiSMASH, and 29 secondary-metabolite biosynthetic gene clusters (BGCs) containing 46 core genes were discovered (Table 2). The core genes, consisting of genes for 25 terpene synthases, 12 nonribosomal peptide synthase enzymes (NRPS-like enzymes), 8 polyketide synthases (PKSs), and 1 indole-related enzyme (Table 2) are distributed on 10 chromosomes (Chr1, -2, -3, -5, -6, -7, -9, -10, -11, and -12) and 5 contigs (ctg15, -17, -24, -25, and -31) (Fig. 5A).

**TABLE 2:** Putative BGCs responsible for secondary metabolites in strain NWAFU-1

Cluster	Location	Start (bp)	Stop (bp)	Core gene ID	Core gene type
1	ctg1	2531802	2548056	g9694.t1	Terpene
2	ctg1	2813581	2835359	g9796.t1	Terpene
3	ctg2	453678	497593	g11207.t1	PKS
4	ctg2	1927623	1943625	g11613.t1	Terpene
5	ctg2	3109735	3161835	g11971.t1	Terpene
				g11978.t1	
				g11982.t1	
6	ctg3	476385	497617	g12368.t1	Terpene
7	ctg5	428286	532709	g4524.t1	NRPS-like
				g4528.t1	PKS
				g4530.t1	NRPS-like
				g4534.t1	NRPS-like
				g4538.t1	PKS
8	ctg6	1102537	1147198	g3837.t1	NRPS-like
9	ctg6	1441185	1442690	g3938.t1	Terpene
10	ctg7	6578	30619	g6728.t1	Terpene
				g6730.t1	
				g6731.t1	
11	ctg7	480774	506133	g6859.t1	Terpene
				g6861.t1	
				g6862.t1	
12	ctg7	594057	615281	g6885.t1	Terpene
13	ctg7	1016899	1064188	g7044.t1	NRPS-like
14	ctg9	183057	203976	g3034.t1	Terpene
15	ctg10	433898	478092	g10146.t1	PKS
				g10149.t1	
16	ctg10	596519	641283	g10195.t1	NRPS-like
17	ctg10	671144	742323	g10219.t1	NRPS-like
				g10220.t1	PKS
				g10229.t1	Terpene
				g10232.t1	
18	ctg11	907664	951621	g13526.t1	PKS
19	ctg11	1162331	1187752	g13618.t1	Terpene
				g13621.t1	
20	ctg11	1418013	1439351	g13710.t1	Terpene
21	ctg11	1484507	1528847	g13738.t1	NRPS-like
22	ctg12	682713	728227	g5863.t1	NRPS-like
23	ctg15	557009	578845	g317.t1	Terpene
24	ctg17	758001	779516	g1290.t1	Indole
25	ctg24	474021	548029	g823.t1	NRPS-like
				g838.t1	NRPS-like
26	ctg25	204719	247787	g10934.t1	PKS
27	ctg25	275478	325614	g10956.t1	PKS
28	ctg31	161801	174725	g571.t1	Terpene
29	ctg31	181167	209940	g581.t1	Terpene
				g582.t1	

Terpenoids, particularly triterpenoids, are the main bioactive constituents of *L. sulphureus*. Among the 25 predicted terpenoid synthases, there are 15 sesquiterpene synthases (STSs) and 8 monoterpene synthases (mono-TPSs), 1 squalene synthase (SQS), and 1 phytoene synthase (PSY) (Table S12). Cluster analysis of NWAFU-1-derived STSs revealed 15 STSs grouped into four clades using known STSs from mushrooms such as Omphalotus olearius (43), Stereum hirsutum (44), and Coprinus cinerea (45). Clade I had the most STSs (6), followed by clade II with four, clade III with three, and clade IV with two (Fig. 5B and Table S13). The structural characteristics of the catalytic products of these STSs can be inferred from the grouping information. The most similar homologies of the eight mono-TPSs are all monoterpene synthase 25 (STS25) from Postia placenta, which catalyzes the cyclization of geranyl diphosphate (GPP) to myristene and linalool (46). STS25 and these 8 mono-TPSs show high similarity, with identifies ranging from 37.78 to 64.95 (Fig. 5C and Table S14). The mevalonate pathway (MVP) is a recognized upstream pathway of fungal terpenoid metabolism. KEGG annotation identifies key genes for MVP in NWAFU-1 (Fig. S9).

Cluster analysis of 9 PKSs and their six most similar homologs (Table S15), as well as LpaA (20), an identified PKS from *L. sulphureus*, showed that four (g10146.t1, g10149.t1, g10956.t1, and g10934.t1) of these PKSs were more closely related to LpaA, but their most similar hits in UniProtKB are not LpaA. The PKS g13526.t1 exhibited 48.52% identify with ArmB (47), an orsellinic acid synthase, and it was speculated that this gene was responsible for the synthesis of orsellinic acid in *L. sulphureus*. The other four PKSs, although showing the most similar hits to Stpks1 in UniProtKB (48), did not point to a usable reference in the current clustering analysis (Fig. 5D).

NRPS-like enzymes other than g4530.t1 all contain at least two functional structural domains (Fig. S9). Further clustering of these NRPS-like enzymes (Table S16) with a total of 46 NRPS-like enzymes (Table S17) identified so far showed that 8 formed a branch with nps9 to -11 and NCU05000. nps9 to -11 were structurally consistent, all containing three structural domains, including an adenylation (A) domain, short-chain dehydrogenase/reductase domain, and thiolation domain. NRPS-like enzymes g5863.t1 and g10129.t1 are in two different clades. In addition to both an A domain and PKS_PP-binding domain, the former contains an NADP_Rossmann domain, while the latter contains a ketide_synthase domain. The remaining NRPS-like enzyme, g7044.t1, did not form clusters, probably because of the additional NUC-45 central domain in its structure (Fig. 5E and Fig. S10).

Cluster 24, containing g1290, was predicted by antiSMASH to be an indole-type BGC, and its core gene, 1290.t1, was annotated as tyrosine dimethylallyl transferase, showing identity with the 4-*O*-dimethylallyl-l-tyrosine synthase TcpD (32%) (49) and SirD (29%) (50). In addition, this BGC contains genes for DUF6534 domain-containing protein (g1287.t1), phenylalanine ammonia-lyase (g1289.t1), flavin adenine dinucleotide (FAD)-binding domain-containing protein (g1291.t1), (4-*O*-methyl)-d-glucuronate–lignin esterase (g1292.t1), and major facilitator superfamily (MFS)-type transporter (g1293.t1) (Fig. 5F). This rare BGC inspired us to explore its widespread distribution, and three similar BGCs derived from Basidiomycetes were found in the NCBI genome database using this BGC as a probe. All three clusters contain at least a core gene (tyrosine dimethylallyl transferase gene) and two modifier genes (phenylalanine ammonia-lyase and FAD-binding domain-containing protein genes) (Fig. 5F). This gene cluster, which uses l-tyrosine as the starting substrate, has been reported only for filamentous fungi so far (49, 50).

To further analyze these BGCs, they were subjected to gene cluster family (GCF) analysis with BIG-SCAPE; the results showed that 4 gene clusters were in the GCF network (Fig. S11). Clusters 15, 26, and 27, all three BGCs containing T1PKS, are all in a complex network of PKS and PKS-NRPS. Clusters 26 and 27 are directly connected, and the similarity analysis of these two also proves this association (Fig. S12A). Cluster 9 is in a GCF network composed entirely of terpenoid BGCs, and the similarity analysis of cluster 9 and the three BGCs directly connected to cluster 9 did not reveal the association (Fig. S12B).

### Cytochrome P450 monooxygenase family analysis.

**FIG 6:**
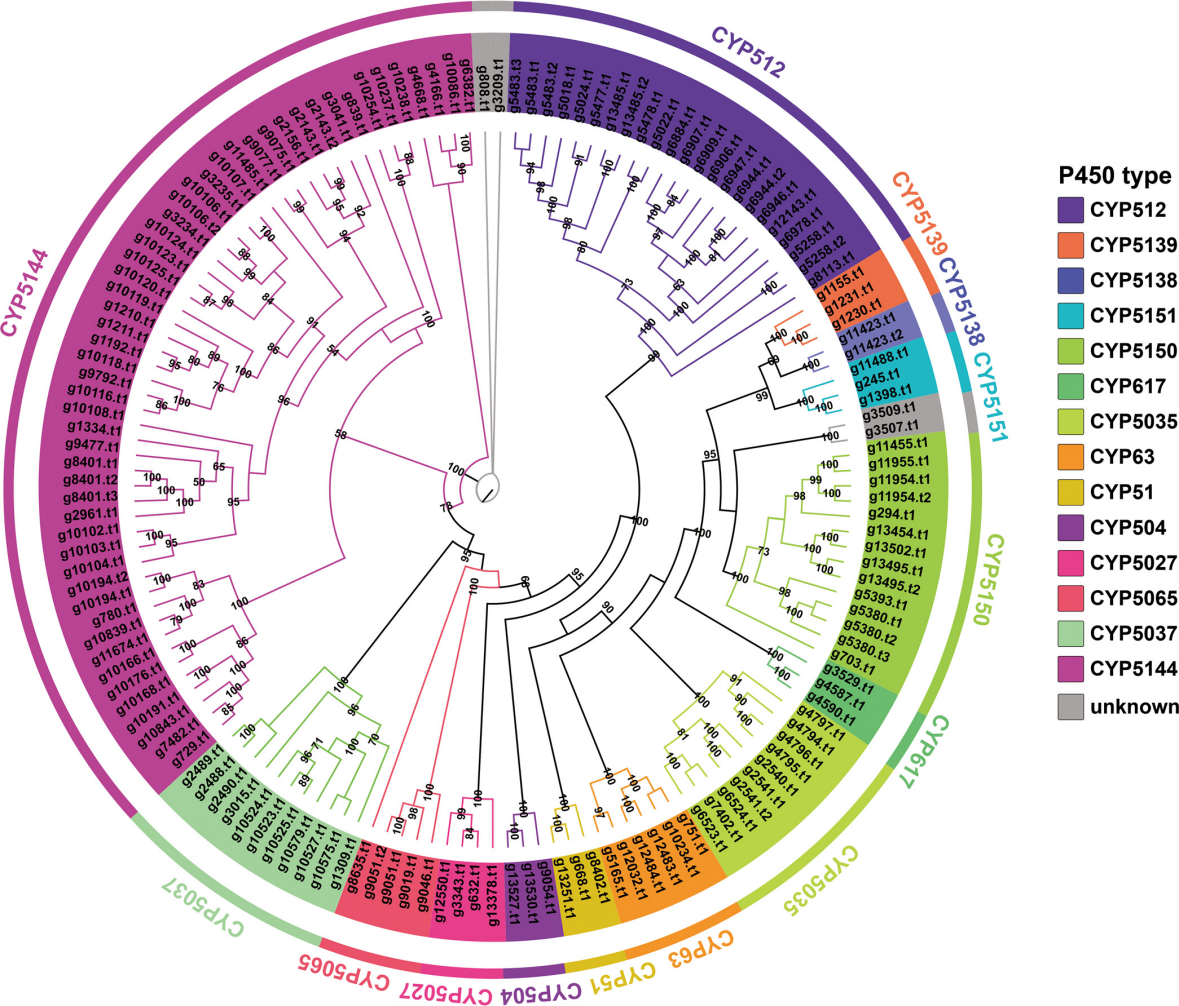
Maximum likelihood method tree of 147 cytochrome P450s from strain NWAFU-1. Each cytochrome P450 family is shown in a separate color, and the branch reliability value of not less than 50 is marked on the corresponding branch node.

Cytochrome P450 is a ubiquitous superfamily of monooxygenases, which are considered to be essential enzymes involved in fungal primary and secondary metabolic processes, including detoxification, exogenous degradation, and biosynthesis of secondary production (51). According to Pfam prediction based on domain features, a total of 130 P450-coding genes (147 P450 proteins) were screened in the genome of strain NWAFU-1 (Table S18). The cluster analysis revealed a clear classification based on the evolutionary association of these 147 protein sequences with the representative basidiomycete P450 sequences of the Fungal Cytochrome P450 Database (Fig. 6, Fig. S13). There were 126 coding genes (143 proteins) identified and divided into 14 cytochrome P450 families, with the CYP5144 family having the most P450s (52), followed by the CYP512 (31), CYP5141 (14), CYP5037 (11), and CYP5035 (10) families. The remaining nine families all have no more than 10 members, accounting for about 16% in total. The P450 family characteristics in *L. sulphureus* are basically consistent with those in Phanerochaete carnosa (Thelephorales) (53).

### Difference and identification of metabolites from fruiting bodies and mycelium.

Most current reports on secondary metabolites of *L. sulphureus* concern the fermentation products of mycelium. In order to investigate whether there are differences in the chemical composition and content of the mycelium and fruiting bodies of *L. sulphureus*, the metabolites from fruiting bodies and the liquid fermentation of the mycelium were used for quantitative high resolution LCMS analysis and compared with GNPS molecular networks. The visualized molecular network showed that the chemical compositions and the contents of the two are significantly different (Fig. S14).

**FIG 7:**
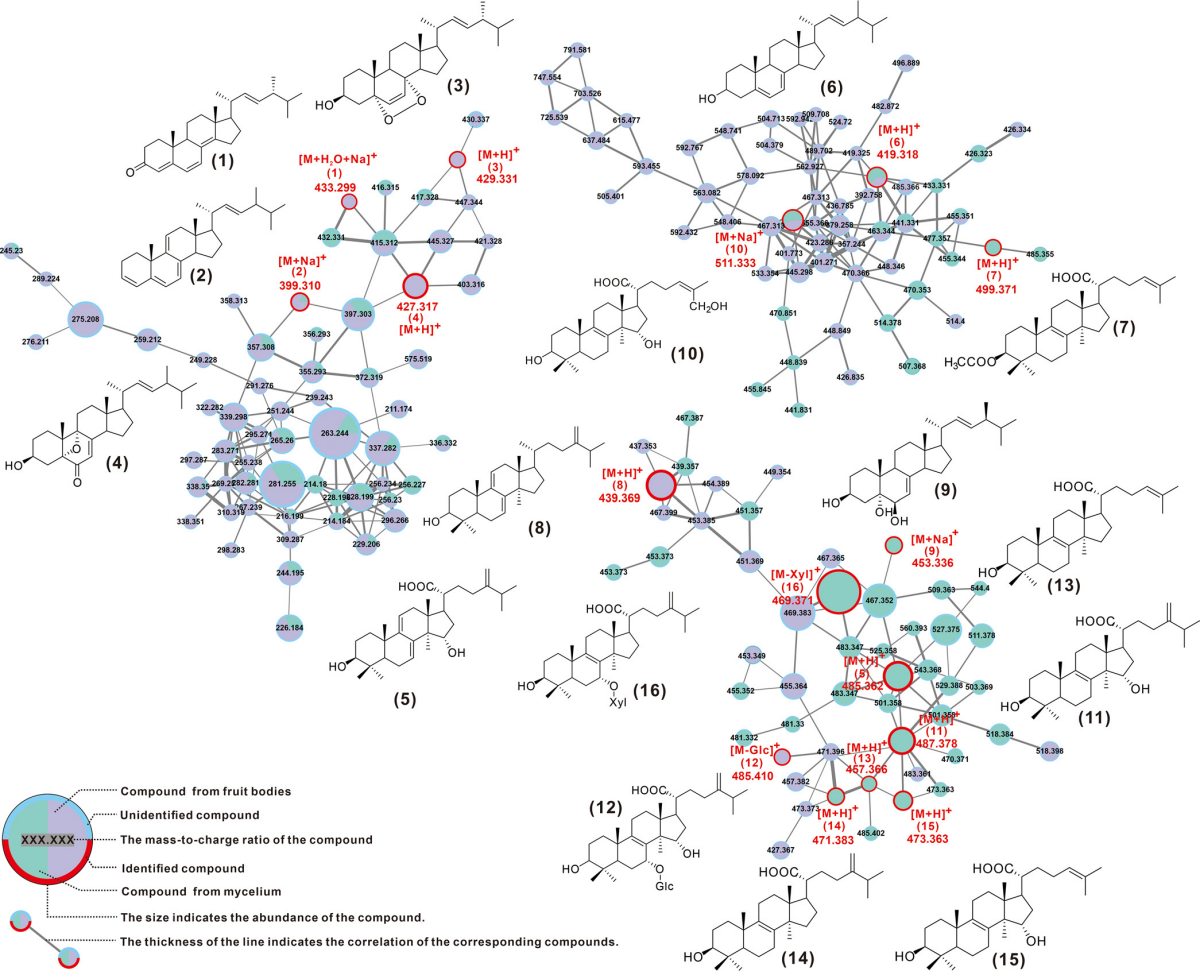
GNPS-based molecular network identification of metabolites.

Further, a total of 16 compounds were identified by comparing their mass spectrometry (MS) and/or nuclear magnetic resonance (NMR) data with reported literature values, including dehydrosulfurenic acid (5), sulfurenic acid (11), tsugaric acid A (12), eburicoic acid (14), laetiposide E (16), trametenolic acid (13) and its derivatives acetyl trametenolic acid (7) and 15α-hydroxytrametenolic acid (15) and other lanostanoids, as well as ergosta-4,6,8(14),22-tetraen-3-one (1), ergost-3,5,7,9(11),22-pentaen (2), ergost-5,7,22-trien-3-ol (6), ergosta-7,22-diene-3,5,6-triol (9), ergosterol peroxide (10), and other ergosteroids (Fig. 7, Table 3, Fig. S15 and S16, and Table S19). Two of the steroids, ergosta-4,6,8(14),22-tetraen-3-one (1) and 3β-hydroxy-5,9-epoxy-(22*E*,24*R*)-ergosta-7,22-dien-6-one (4), are reported for the first time for the genus *Laetiporus*. Except for compound 2, which was present in both mycelium and fruiting body, the other compounds were only distributed in one of the two tissues, which reflected the tissue specificity of the distribution of basidiomycete-derived compounds.

**TABLE 3:** Identified metabolites from strain NWAFU-1

No.	Source	Putative metabolite	Molecular formula	Adduct	*m/z*	Reference
1	Fruiting body	Ergosta-4,6,8(14),22-tetraen-3-one	C_28_H_40_O	[M+Na+H_2_O]^+^	415.2977	52
2	Fruiting body and mycelium	Ergost-3,5,7,9(11),22-pentaen	C_28_H_40_	[M+Na]^+^	377.3130	62
3	Fruiting body	5α,8α-epidioxy-(22*E*,24*R*)-ergosta-6,22-dien-3b-ol	C_28_H_44_O_3_	[M+H]^+^	429.3290	63
4	Fruiting body	3β-Hydroxy-5,9-epoxy-(22*E*,24*R*)-ergosta-7,22-dien-6-one	C_28_H_42_O_3_	[M+H]^+^	427.3207	64
5	Fruiting body	Dehydrosulfurenic acid	C_31_H_48_O_4_	[M+H]^+^	485.3625	65
6	Mycelium	Ergost-5,7,22-trien-3-ol	C_30_H_48_O	[M+H]^+^	425.3778	62
7	Mycelium	Acetyl trametenolic acid	C_32_H_50_O_4_	[M+H]^+^	499.3782	66
8	Fruiting body	24-methylenelanost-7,9-dien-3-ol	C_31_H_50_O	[M+H]^+^	439.3934	62
9	Fruiting body	Ergosta-7,22-dien-3,5,6-triol	C_28_H_46_O_3_	[M+Na]^+^	431.3520	62
10	Mycelium	Ergosterol peroxide	C_28_H_46_O_3_	[M+Na]^+^	453.3339	62
11	Mycelium	Sulfurenic acid	C_31_H_50_O_4_	[M+Na]^+^	509.3601	67
12	Mycelium	Tsugaric acid A	C_31_H_50_O_4_	[M+H]^+^	487.3782	66
13	Mycelium	Trametenolic acid	C_30_H_48_O_3_	[M+H]^+^	457.3676	67
14	Mycelium	Eburicoic acid	C_31_H_50_O_3_	[M+H]^+^	471.3833	67
15	Mycelium	15α-Hydroxytrametenolic acid	C_30_H_48_0_4_	[M+H]^+^	473.3625	67
16	Fruiting body	Laetiposide E	C_36_H_58_O_8_	[M+H]^+^	619.4310	67

### Speculation on the biosynthesis of lanostanoids derived from *L. sulphureus*.

**FIG 8:**
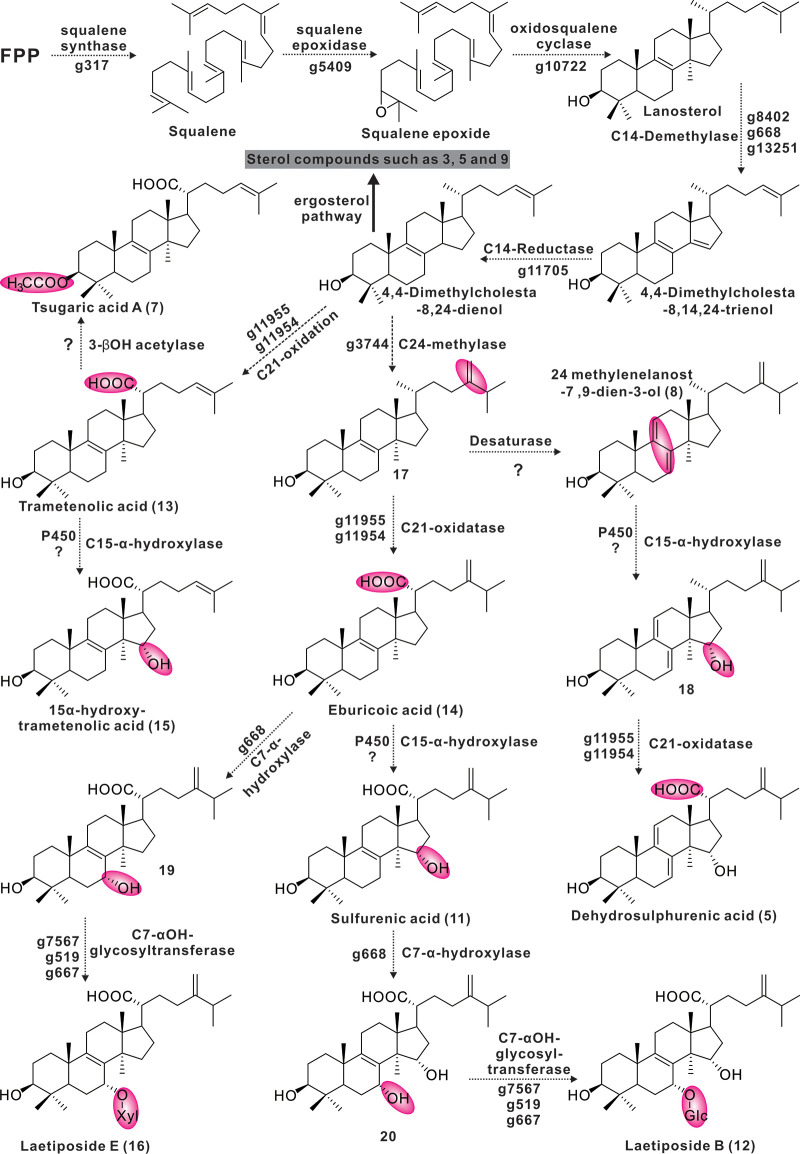
The proposed biosynthetic pathway of tetracyclic triterpenes from *L. sulphureus*.

Structurally, the 16 identified compounds can be divided into two classes, lanostanoids and ergosteroids. Based on what is known, it is assumed that these compounds are all derivatives of the lanosterol-to-ergosterol pathway, with 4,4-dimethylcholestta-8,14,24-trienol acting as a point of divergence (Fig. 8). Enzymes related to the identified ganoderic acid biosynthesis enzymes responsible for the pathway of farnesyl pyrophosphate to lanosterol (54) and the corresponding homologous genes were highly similar. Three candidate genes (g8402, g668, and g13251) responsible for the conversion of lanosterol to 4,4-dimethylcholestta-8,14,24-trienol were identified, and the corresponding three cytochrome P450s are members of the CYP51 family (Fig. 6). The protein encoded by g11705 is responsible for the C-14 reduction of 4,4-dimethylcholestta-8,14,24-trienol, which is very similar to the sterol C-14 reductase from *Phellinus igniarius* (Table S20). Trametenolic acid (13) is formed by the sequential oxidation of C-21 of 4,4-dimethylcholestta-8,24-dienol, which is presumably accomplished by g11955 or g11954. Its protein is highly like CYP5150L8, an enzyme responsible for a similar process in the formation of ganoderic acid 3-hydroxy-lanosta-8,24-dien-26-oic acid (54). Compound 15 is formed by α-hydroxylation of C-15 on compound 13, which is presumably also carried out by cytochrome P450. Tsugaric acid A (12) is produced by methylating the C-4 of compound 15. The 3-hydroxyl group of compound 13 is acetylated to form compound 8. The presumed intermediate compound 17 is produced by C-24 methylation of 4,4-dimethylcholestta-8,24-dienol, which opens another pathway. Methylation of C-24 is thought to be the responsibility of the homolog of ERG6, g3744, and the two proteins catalyze very similar reactions. C-7 of eburicoic acid (14) undergoes α-hydroxylation and then glycosylation to form Laetipides E (16). The methylation of the C-7 position of this tetracyclic backbone (sterol) is rarely reported, such as in the cases of Homo sapiens (55) and Rattus norvegicus (56), and the results of genome scanning with the corresponding proteins showed that g668 was the best-matching hit (Fig. S17). It is speculated that g668 is responsible for the former step, and there are three candidate genes (g7567, g519, and g667) for the latter step. α-Methylation of C-15 of compound 14 resulted in a shunting metabolism to form eburicoic acid (11) (Fig. 8). The network in which eburicoic acid (16) is found contains all three putative intermediate compounds 17 to 19 (Fig. S18 and Table S20).

## DISCUSSION

The fungi of the family Polyporaceae, represented by *G. lucidum* and *Inonotus obliquus*, are significant complementary and alternative medicines as well as ancient traditional medicines that have been proven to have significant health-promoting effects. Ganoderic acids, lanosterol tetracyclic triterpenoids from *G. lucidum*, have been proven by modern medicine to have medicinal value, and their biosynthesis has been extensively analyzed (57). Eburicoic acid, sulfurenic acid, and their derivatives are lanosterol tetracyclic triterpenoids derived from *L. sulphureus* and have also been reported for other Polyporaceae fungi (11, 16). Such functional molecules have been shown to have antitumor, analgesic, anti-inflammatory, and hepatoprotective effects (11, 16, 58, 59). The present work provides detailed speculation on the biosynthesis of these metabolites and identifies the candidate genes responsible for transformation processes. This investigation provides a clear baseline for subsequent experiments to validate these processes.

The active ingredients of many ancient medicinal macrofungi are commonly derived from the fruiting bodies, and prescriptions in medical classics also emphasize that the medicinal parts are fruiting bodies. In fact, it is not easy for medicinal fungi to form seeds in their natural environment, which explains the rarity of large medicinal fungi. The lack of available sources of fruiting bodies is a major limitation to the large-scale medicinal application of macrofungi. A viable solution to this limitation is artificial cultivation (2). Although there has been a report claiming to have achieved artificial cultivation of *L. sulphureus*, the mating type of *L. sulphureus* has not yet been determined. The present work investigated the mating type of *L. sulphureus* based on genomic information and found that the MAT-A locus on chromosome 13, far from the MAT-B locus on ctg17, was unrelated, and it is reasonable to conclude that *L. sulphureus* has a tetrapolar mating system. This conclusion will provide valuable clues for the efficient large-scale cultivation of *L. sulphureus*.

The genome sequencing, assembly, and functional annotation of macrofungi provide valuable information for understanding the genetic background and studying gene function and can provide a theoretical basis for the construction of genetic manipulation systems, molecular breeding, and high-yield cultivation. In this study, the whole-genome sequencing and analysis of *L. sulphureus* NWAFU-1 showed that this strain was rich in CAZymes and had the potential to synthesize various secondary metabolites, especially in terms of the highly enriched polysaccharide and terpenoid biosynthesis genes. Comparative genomic analysis showed that strain NWAFU-1, unlike other sequenced subspecies, underwent a larger genome duplication event. Based on the molecular network analysis of metabolites and genome scanning, it was speculated that the active triterpenoids originated from the lanosterol pathway, and several potential genes of the transformation process were pointed out. These findings provide important insights into the biological characteristics of the medicinal edible fungus *L. sulphureus*, including growth characteristics and biosynthetic pathways of active compounds. In addition, this study is expected to provide basic information for the development of mushroom genomes and germplasm resources, to provide a reference for elucidating the biosynthetic mechanisms of various secondary metabolites in medicinal edible fungi, and possibly to promote synthetic biology research in mushrooms.

## MATERIALS AND METHODS

### Microbial strains and culture conditions.

*L. sulphureus* NWAFU-1 was isolated from the bark of lacquer tree (*Toxicodendron vernicifluum*) in the Qinling Mountains. The strain was stored in Shaanxi Province Key Laboratory of Chemical Biology & Natural Products. Solid cultures of the strains were carried out on potato dextrose agar medium at 25°C, and liquid cultures of the strains were accomplished in shake flasks containing potato dextrose broth medium at 200 rpm and 25°C.

### Genome sequencing and assembly.

The genomic DNA was extracted from mycelia with a Covaris ultrasonic crusher. The sample then underwent end repair, addition of an A tail, addition of sequencing connectors, purification, and PCR amplification. High-quality bulk DNA was collected to build the library after checking purity, concentration, and integrity. Qubit 2.0 was used for preliminary quantification to detect whether the inserted fragment of the library met expectation before the concentration of the library was accurately quantified by quantitative PCR to ensure the quality of the library. Then, the prepared library was pooled to flow cell, and Illumina NovaSeq was used for sequencing after clustering. The Oxford Nanopore PromethION sequenator was supported by the software Guppy to automatically distinguish between pass and fail data. After removal of reads with an N base content exceeding 5%, reads with 50% of bases in low quality (mass value less than or equal to 5), reads contaminated with adapters, and the repeat sequence caused by PCR amplification, a total of 79,622,624 clean reads and 5,582,739,300 bases were generated. The genome size of 47.7 Mb was estimated by the k-mer method using sequencing data from the DNA library. NECAT software (https://github.com/xiaochuanle/NECAT) was used to perform genome error correction, and splicing was performed to obtain the initial splicing result; then, Racon (https://github.com/isovic/racon) 1.4.11 software was used to perform two rounds of error correction on the splicing result based on the third-generation sequencing data, and, finally, two rounds of Pilon were performed (v.1.23). Error correction was performed, and after removal of heterozygosity, the final assembly result was obtained. BUSCO 4.1.4 software (https://github.com/RoyNexus/busco) evaluated 93.3% of the predicted genes integrity based on the fungal database (fungi_odb10) (v.4.0.6), showing an excellent assembly level. The mitochondrial genome reads were extracted from the whole-genome data by bowtie, and the mitochondrial genome was assembled using GeSeq, which performed annotation using MITOS and GeSeq, based on genetic code 4. Genome collinearity analysis and visualization were achieved by McscanX (https://opensourcelibs.com/lib/mcscanx).

### Gene prediction and annotation.

Gene prediction was performed mainly using BRAKER software (v.2.1.4); first, GeneMark-EX was used to train the model, and then, AUGUSTUS was called for prediction. INFERNAL (v.1.1.2) was used to predict and classify ncRNA based on the Rfam database. RepeatModeler software (v. 1.0.4) was used to build its own repeat library, and RepeatMasker (v.4.0.5) was used to annotate the repeated sequence of the genome after merging the repbase library. Functions of the gene products were annotated based on BLAST searches of nonredundant protein sequences from the NCBI, Swiss-Prot, COG, and KEGG databases. GC-Profile characterizes changes of GC content in genomic sequences and predicts genomic islands, which were generated on a web-based tool. Nr annotation showed that strain NWAFU-1 shared 95% genes with *L. sulphureus* 93-53.

### Phylogenomic and comparative genomic analyses.

Homologous gene identification and phylogenetic analysis were performed with strain NWAFU-1 and another a total of 21 representative strains in Russulales, Agaricales, and Hymenochaetales. Single-copy homologous genes were identified using OrthoFinder (v.2.5.4) (60) with the parameters as “-S diamond -M msa -T raxml-ng.” MCMCtree (http://abacus.gene.ucl.ac.uk/software/paml.html) was utilized to predict divergence time with a total of 520 single-copy ortholog sequences of 24 strains. Several groups of recent ancestor divergence times were queried as calibrated points in TimeTree (http://www.timetree.org/) (*H. irregulare* versus *S. hirsutum*, 71.3 to 125.1 MYA; Phanerochaete sordida versus Phlebiopsis gigantea, 65.3 to 103.7 MYA; and *G. sinense* versus Dichomitus squalens, 18.2 to 34.7 MYA).

Comparative genomic analysis within *L. sulphureus* subspecies was performed through single-copy genes and visualized by jVenn (http://jvenn.toulouse.inra.fr/app/index.html). *K_s_* calculation was performed among three *L. sulphureus* subspecies. The homolog protein sequence identifier (ID) lists were transformed to CDS lists by ParaAT 2.0 (https://github.com/wonaya/ParaAT). Homologous sequence pairs among the three subspecies were calculated by KaKs Calculator 3.0 (https://ngdc.cncb.ac.cn/biocode/tools/BT000001) and finally visualized by Rstudio (v. 4.2.0).

### CAZy family analysis.

The database CAZy (http://bcb.unl.edu/dbCAN2/) was used to annotate and class the genes encoding CAZymes from NWAFU-1 and other brown-rot Basidiomycota, with HMMER (v.3.2.1; filter parameter E value < 1e^−5^; coverage > 0.35).

### Prediction and phylogenetic analyses of gene clusters involved in secondary metabolites.

The antibiotic and secondary-metabolite production gene clusters were examined using antiSMASH 6.1 software and FramePlot with default parameters. To verify the predicted results, the obtained gene clusters were manually checked. BLASTP analysis and gene annotation were performed using the NCBI genome portal software platform. We searched all hypothetical gene models in the database using blastP and tblastN algorithms. BIG-SCAPE was performed to build a BGC network between BGCS predicted by antiSMASH (https://antismash.secondarymetabolites.org/) and confirmed BGCs in the MiBIG1.4 database with a cutoff of 0.75, and the BGC network was visualized by Cytoscape3.9.1. Farnesyl diphosphate synthase (FPPs) were selected from terpene synthases predicted by antiSMASH by 2ndFind for further analysis. The FPPs phylogenetic tree showed a clear classification. Prediction for PKS domains was analyzed by PKS/NRPS analysis program online (http://nrps.igs.umaryland.edu/tutorial.html). For PKS BGCs, the KS domains of *L. sulphureus* and related species were collected from the NCBI and JGI database to perform phylogenetic analysis to generate a maximum likelihood tree using OrthoFinder software to describe its evolutionary status. For NRPS-like BGCs, all the domains of *L. sulphureus* and other species that had been identified in UniProt (https://www.uniprot.org) were collected to generate a basic phylogenetic tree to understand the polygenetic status. Comparison of indole-type BGCs with similar BGCs was achieved by clinker (61).

### Prediction and analysis of cytochrome P450s.

The package Hmmer was used to predict cytochrome P450s with Diamond (version > 2.9.0; E value > e^−5^) and annotate the target protein sequence. The reference CYP sequences for cluster analyses were downloaded from the Fungal Cytochrome P450 Database (http://p450.riceblast.snu.ac.kr/index.php?a=view). A total of 147 cytochrome P450 proteins of strain NWAFU-1 and several other similar species selected from the fungal cytochrome P450 database were clustered to perform phylogenetic tree analysis with clear classification. A maximum likelihood tree was built by IQ-tree 2.2.3 with parameters “-m MFP -bb 1000 -alrt 1000 -abayes -nt AUTO.”

### Metabolite analysis and structural evaluation.

To analyze the metabolites of strain NWAFU-1, liquid fermentation and solid fermentation were performed; the solid fermentation was performed on PDA medium at 25°C for 14 days. Liquid fermentations were grown in shake flasks with PDB at 200 rpm and 25°C for 10 days. After fermentation was complete, the cultures were harvested and extracted 3 times with an equal volume of ethyl acetate to obtain as many secondary metabolites as possible. The bioactive metabolite extracts and their fractions were analyzed using high-resolution electrospray ionization mass spectrometry (HRESIMS) was carried out using AB Sciex TripleTOF 6600 mass spectrometer. The electrospray mass spectra were recorded on a time-of-flight mass spectrometer in both positive-ion and negative-ion modes. One-dimensional nuclear magnetic resonance (1D-NMR) data were obtained with a Bruker (Germany) ADVANCE IIIHD 400-MHz NMR spectrometer including ^1^H NMR and ^13^C NMR. The structures of the isolated compounds were established based on spectroscopic analyses and comparisons to previously reported data sets. Molecular network analysis of high-performance liquid chromatography (HPLC)–HRESIMS data of the crude extract was performed by GNPS with default parameters. The network file based on negative-ion-mode MS data can be found and accessed at https://gnps.ucsd.edu/ProteoSAFe/status.jsp?task=ec420f568ec64f7d82470a7cf507b35f. The molecular network was visualized by Cytoscape 3.9.1. The compounds identified by NMR and HRMS are in the same network and show a good correlation with analogs with similar structure.

### Data availability.

The ITS sequence of strain *L. sulphureus* NWAFU-1 has been deposited in the NCBI GenBank under accession number ON797482. The final genome assembly results and related data of *L. sulphureus* NWAFU-1 have been submitted to the National Center for Biotechnology Information (NCBI) under the BioProject JAMXVQ000000000 and BioSample SAMN29145082, respectively. For details, see Data Set S1 (gene distribution of 22 brown-rot fungi based on the six major modules of CAZymes), Data Set S2 (the protein sequences of the predicted genes for *Laetiporus sulphureus* NWAFU-1), Data Set S3 (the DNA sequences of the predicted genes for *Laetiporus sulphureus* NWAFU-1), and Data Set S4 (the chromosome where the predicted gene is located and its specific location on the chromosome).
