# Supplementary material for: Chromosome-Level Genome Sequences, Comparative Genomic Analyses, and Secondary-Metabolite Biosynthesis Evaluation of the Medicinal Edible Mushroom Laetiporus sulphureus
Source: Microbiol Spectr. 2022 Oct 6;10(5):e02439-22. doi: 10.1128/spectrum.02439-22 (PMC9602373; doi:10.1128/spectrum.02439-22)
Supplement: Supplemental file 1 — Fig. S1 to S18 and Tables S1 to S20. Download spectrum.02439-22-s0001.pdf, PDF file, 6.2 MB [file spectrum.02439-22-s0001.pdf]

Chromosome-Level Genome Sequences, Comparative Genomic Analyses, and Secondary Metabolite Biosynthesis Evaluation for the Medicinal-Edible Mushroom *Laetiporus. sulphureus*

Wei-ge Dong [a#], Zhen-xin Wang [a#], Xi-long Feng[a], Rui-qi Zhang[a], Dao-yin Shen[b], Shuangtian Du[a], Jin-ming Gao[a] Jianzhao Qi[a]*

[a] Shaanxi Key Laboratory of Natural Products & Chemical Biology, College of Chemistry & Pharmacy, Northwest A&F University, 3 Taicheng Road, Yangling 712100, Shaanxi, China

[b] Yangling Zhijun Fungi Biotechnology Engineering Co., Ltd., Modern Agricultural Incubation Park, Nongke Road, Yangling 712100, Shaanxi, China

# These authors contributed equally to this paper.

*Corresponding author (Tel: +86-29-87092381 E-mail: qjz@nwafu.edu.cn)

Content

Table S1. Statistics of Oxford Nanopore PromethION sequencing data volume of *L. sulphureus* NWAFU-1 genome. 1
Table S2. Statistics of Illumina NovaSeq sequencing data volume information of *L. sulphureus* NWAFU-1 genome. 2
Table S3. Statistics of Illumina NovaSeq sequencing data mapping of *L. sulphureus* NWAFU-1 genome. 3
Table S4. Statistics of BUSCO evaluation of *L. sulphureus* NWAFU-1 genome. 4
Table S5. Annotation Statistics of coding gene from the *L. sulphureus* NWAFU-1 genome. 5
Table S6. Statistics of non-coding RNA annotation results in *L. sulphureus* NWAFU-1 genome. 6
Table S7. Statistics of *L. sulphureus* NWAFU-1 repetitive sequence annotation results. 7
Table S8. Statistics of *L. sulphureus* NWAFU-1 protein-coding gene annotation. 8
Table S9. The candidate genes for mating type in *L. sulphureus* NWAFU-1 genome. 9
Table S10. The source (URL) statistics for 22 brown-rot fungi used for CAZymes analysis. 10
Table S11. The polysaccharide biosynthetic candidate genes in *L. sulphureus* NWAFU-1 genome. 11
Table S12. Terpenoid biosynthesis related enzymes in *L. sulphureus* NWAFU-1 genome. 12
Table S13. Identified sesquiterpenes derived from Basidiomycetes used to construct sesquiterpene phylogenetic tree. 13
Table S14. Identify matrix between 8 STSs from NWAFU-1 and STS25. 14
Table S15. Sequence homology analysis for 9 PKSs from the strain NWAFU-1. 15
Table S16. Sequence homology analysis for 11 NRPS-likes from the strain NWAFU-1. 16
Table S17. 46 identified NRPS-likes used in cluster analysis with 11 NRPS-Likes. 17
Table S18. Identification of cytochrome P450 genes in *L. sulphureus* NWAFU-1 genome. 18
Table S19. The NMR data of three compounds from the strain NWAFU-1 22
Table S20. The candidate genes related to Tetracyclic triterpene derivatives synthesis in *L. sulphureus* NWAFU-1 genome. 23
Figure S1. ITS alignment of the strain NWAFU-1. 24
Figure S2. Kmer-Depth and Kmer Species-Frequency Distribution Plot. 25
Figure S3. Species distribution map of Nr database alignment to sequences. 26
Figure S4. Statistical map of functional annotation classification based on GO database 27
Figure S5. Statistical Chart of COG Functional Annotated Classification. 28
Figure S6. KEGG Pathway Functional Classification Chart. 29
Figure S7. Domain annotation based on the Pfam database. 30
Figure S8. Comparison of the similarity of related genes on the matA and matB loci of NWAFU-1 and four mushroom species. 31
Figure S9. Annotation of the terpenoid backbone biosynthetic pathway of NWAFU-1 using KAAS. 32
Figure S10. Domain analysis of 11 NRPS-like enzymes from the genome of the strain NWAFU-1. 33
Figure S11. GCF analysis of BGCs from the strain NWAFU-1. 34
Figure S12. BGC similarity analysis of clusters 26 and 27(A), and 9 and others (B). 35
Figure S13. P450s Cluster analysis of the strain NWAFU-1 and other Basidiomycete. 36
Figure S14. Molecular network analysis of metabolites from the mycelium and fruiting bodies of the strain NWAFU-1 37
Figure S15. The LC-ESI-HRMS and LC-ESI-HRMS/MS spectrums of isolates from the strain NWAFU-1. 39
Figure S16. The NMR spectrums of compounds (1, 4, 5, and 11) from the strain NWAFU-1. 41
Figure S17. Genome scanning of NWAFU strains was performed with C7 hydroxylases derived from *Homo sapiens* (A) and *Rattus norvegicus* (B). 42
Figure S18. Identification of putative intermediates (17-20) by molecular network (A) and HR-MS(B-E)... 43
Reference 44

Table S1. Statistics of Oxford Nanopore PromethION sequencing data volume of *L. sulphureus* NWAFU-1 genome.

Rank	Flag	TotalBase	TotalReads	MaxLen	AvgLen	N50	L50	N90	L90	meanQ
>0	all	11,941,826,471	3,283,862	1,150,818	3,636.51	14,110	195,829	1,385	1,496,689	11.45
>0	pass	11,524,053,542	3,183,399	617,996	3,620.04	13,913	192,149	1,379	1,4571656	11.61
>0	fail	417,772,929	100,463	1,150,818	4,158.47	20,713	4,073	1,577	39,382	6.15
>5000	all	8,061,373,665	455,432	1,150,818	17,700.49	26,534	112,826	7,452	321,877	11.25
>5000	pass	7,754,979,809	441,208	617,996	17,576.69	26,329	108,632	7,435	312,589	11.42
>5000	fail	306,393,856	14,224	1,150,818	21,540.62	34,140	4,195	7,992	9,307	5.93
>10000	all	6,696,462,646	256,944	1,150,818	26,061.95	31,202	81,447	13,766	199,840	11.23
>10000	pass	6,434,419,212	249,175	617,996	25,822.89	30,949	78,445	13,731	194,243	11.40
>10000	fail	262,043,434	7,769	1,150,818	33,729.36	40,951	3,002	14,838	5,613	5.82
>50000	all	1,270,629,315	19,772	1,150,818	64,264.07	60,870	913	51,720	17,274	11.08
>50000	pass	1,160,757,043	18,855	617,996	61,562.29	59,958	870	51,601	16,570	11.38
>50000	fail	109,872,272	917	1,150,818	119,817.09	158,538	43	55,898	710	5.08
>100000	all	88,698,477	445	1,150,818	199,322.42	222,052	3	106,251	359	6.39
>100000	pass	19,640,223	163	617,996	120,492.16	111,231	2	101,435	144	10.14
>100000	fail	69,058,254	282	1,150,818	244,887.42	297,083	2	121,533	220	4.22

Rank is the gradient of data length, **>0** is that all data; **Flag** is data type, **all** is all sequencing data, **pass** is effective sequencing data, **fail** is filtration data; **TotalBase** is the number of base; **TotalReads** is the number of reads; **MaxLen** is maximum length of data; **AvgLen** is average length of data; **N50** is N50 of data, all reads are summed in order from long to short, and when additive length up to half of all reads total length, the last read length added is N50; **L50** is L50 of data, all reads are accumulated in turn ranking in order from long to short, when additive length up to half of all reads total length, the number of sequences is L50; **N90** is N90 of data, algorithm the same as N50; **L90** is L90 of data, algorithm the same asL50; **meanQ** is mean quality value.

Table S2. Statistics of Illumina NovaSeq sequencing data volume information of *L. sulphureus* NWAFU-1 genome.

Sample_name	Total_reads	Total_bases	GC_content	Q20	Q30
rawdata	79,622,624	11,943,393,600	50.54%	97.89%	93.90%
cleandata	79,622,624	11,943,393,600	50.54%	97.89%	93.90%

Sample name is data type; **Total_reads** is sequencing reads number; **Total_bases** is total sequencing base number; **GC_Content** is G/C base number as a percentage of total base number; **Q20、Q30** respectively are Phred value greater than 20、30 base as a percentage of total base.

Table S3. Statistics of Illumina NovaSeq sequencing data mapping of *L. sulphureus* NWAFU-1 genome.

**Type**	**Number**
map_rate	88.49%
Average_depth	214.04
Coverage	99.81%

map_rate is Illumina NovaSeq sequencing data mapping rates; **Average_depth** is average coverage depth; **Coverage** is coverage rate.

Table S4. Statistics of BUSCO evaluation of *L. sulphureus* NWAFU-1 genome.

Item	Number	Percent (%)
Complete BUSCOs (C)	707	93.3
Complete and single-copy BUSCOs (S)	631	83.2
Complete and duplicated BUSCOs (D)	76	10.0
Fragmented BUSCOs (F)	16	2.1
Missing BUSCOs (M)	35	4.6
Total BUSCO groups searched	758	100.0

Table S5. Annotation Statistics of coding gene from the *L. sulphureus* NWAFU-1 genome.

Type	Number
Total number of gene	15,302
Average of mRNA length	1,740.93
Average of cds length	1,272.37
Average of exon number	7.27
Average of exon length	174.94
Average of intron length	74.68
Total number of exon	111,297
Total number of intron	95,995
Total intron length	7,169,351

the total number of gene is the total number of genes; the average of mRNA_length is the average length of mRNA; the average of cds_length is the average length of CDS; the average of exon_number is the average number of exons per gene. the average of exon_length is the average exon length; the average of intron_length is the average intron length; the total number of exon is the total number of exons; the total number of intron is the total number of introns; the total intron length is the total intron length.

Table S6. Statistics of non-coding RNA annotation results in *L. sulphureus* NWAFU-1 genome.

Class	number	totalLen(bp)	meanLen(bp)
rRNA	50	65,477	1,309
sRNA	1	346	346
snRNA	19	2,396	126
tRNA	81	6,957	85

rRNA is ribosomal RNA; tRNA is transport RNA; sRNA is small regulatory RNA; snRNA is nucleolar small RNA. **totalLen** and **meanLen** are the total length and mean length.

Table S7. Statistics of *L. sulphureus* NWAFU-1 repetitive sequence annotation results.

Item	Subfamily	Number	Length(bp)	Coverage
SINE	/	4	295	0.00%
LINE	/	257	126,230	0.26%
LTR	/	1,835	2,383,754	4.93%
LTR	Gypsy	1,229	1,970,733	4.08%
LTR	Copia	495	398,941	0.83%
DNA	/	1,024	729,954	1.51%
Satellite	/	25	3,176	0.01%
Simple_repeat	/	2,917	123,585	0.26%
Low_complexity	/	450	22,894	0.05%
Other	/	145	329,735	0.68%
Unknown	/	10,006	5,483,956	11.35%
Total	/	16,663	9,067,448	18.76%

SINE is short scattered element; **LINE** is Long scattered element; **LTR** is long terminal repetition, mainly include two types, Gypsy and Copia; **DNA** is transposons; **Satellite** is satellite repetitive sequence; **Low_complexity** is Low_complexity repetition; **Other** is other types repetition; **Unknown** is unknown repetitive sequence; **Total** is total repetitive sequence.

Table S8. Statistics of *L. sulphureus* NWAFU-1 protein-coding gene annotation.

Item	Count	Percentage
All	15,302	100%
Annotation	13,380	87.44%
Uniprot	6,739	44.04%
Pfam	9,523	62.23%
Refseq	4,655	30.42%
Nr	13,307	86.96%
Interproscan	9,558	62.46%
GO	6,689	43.71%
KEGG	5,010	32.74%
Pathway	3,064	20.02%
COG	1,350	8.82%

Annotation is the gene with at least one annotation; Uniprot is the gene annotated to the Uniprot database; Pfam is the gene that is annotated to the Pfam database; Refseq is the gene that is annotated to the Refseq database; Nr is the gene that is annotated to the Nr database; Interproscan is the gene that is annotated to the Interproscan GO is the gene annotated to the GO database; KEGG is the gene that is annotated to the KEGG database; Pathway is the gene that is annotated to the KEGG Pathway database; COG is the gene that is annotated to the COG database.

Table S9. The candidate genes for mating type in *L. sulphureus* NWAFU-1 genome.

Top Annotation	Gene ID	Species	E -Value	Accession Number
mitochondrial intermediate peptidase (*mip*)	g6577<br>g6579	*Amylocystis lapponica*<br>*Phanerochaete carnosa*	0<br>$3e^{-39}$	KAH9932403.1<br>XP_007402198.1
homeodomain transcription factors (*HD*)	g6581<br>g6582	*Antrodia cinnamomea*<br>*Antrodia serialis*	$1e^{-34}$<br>$8e^{-09}$	KAI0936809.1<br>XP_047900381.1
glycosyltransferase family 8 protein (*glgen*)	g6685	*Rhodofomes roseus*	$2e^{-150}$	KAH9835885.1
beta-flanking gene (*β-fg*)	g6583	*Antrodia cinnamomea*	$7e^{-48}$	KAI0964037.1
STE3-like pheromone receptor (*ste3*)	g1249<br>g1253	*Antrodia cinnamomea*<br>*Antrodia serialis*	$1e^{-104}$<br>$1e^{-169}$	KAI0920336.1<br>XP_047884634.1

Table S10. The source (URL) statistics for 22 brown-rot fungi used for CAZymes analysis.

**Species**	**Source**
L. sulphureus NWAFU-1	in the study
Calocera cornea	https://www.ncbi.nlm.nih.gov/genome/?term=Calocera%20cornea
Calocera viscosa	https://www.ncbi.nlm.nih.gov/genome/?term=Calocera+viscosa
Coniophora puteana	https://www.ncbi.nlm.nih.gov/genome/?term=Coniophora+putean
Daedalea quercina	https://www.ncbi.nlm.nih.gov/genome/?term=Daedalea+quercina
Fistulina hepatica	https://www.ncbi.nlm.nih.gov/genome/?term=Fistulina+hepatica
Fomitopsis betulina	https://www.ncbi.nlm.nih.gov/genome/?term=Fomitopsis+betulina
Fomitopsis pinicola	https://www.ncbi.nlm.nih.gov/genome/?term=Fomitopsis+pinicola
Fomitopsis rosea	https://www.ncbi.nlm.nih.gov/genome/?term=Fomitopsis+rosea
Gloeophyllum trabeum	https://www.ncbi.nlm.nih.gov/genome/?term=Gloeophyllum+trabeum
L. sulphureus 93-53	https://www.ncbi.nlm.nih.gov/genome/?term=L.+sulphureus
Neolentinus lepideus	https://www.ncbi.nlm.nih.gov/genome/?term=Neolentinus+lepideus
Paxillus ammoniavirescens	https://www.ncbi.nlm.nih.gov/genome/?term=Paxillus+ammoniavirescens
Paxillus involutus	https://www.ncbi.nlm.nih.gov/genome/?term=Paxillus+involutus
Paxillus rubicundulus	https://www.ncbi.nlm.nih.gov/genome/?term=Paxillus+rubicundulus
Poria cocos	https://www.ncbi.nlm.nih.gov/genome/?term=Poria+cocos
Postia placenta	https://www.ncbi.nlm.nih.gov/genome/?term=Postia+placenta
Pycnoporellus fulgens	https://genome.jgi.doe.gov/portal/pages/dynamicOrganismDownload.jsf?organism=Pycful1
Rhodofomes roseus	https://www.ncbi.nlm.nih.gov/genome/?term=Rhodofomes+roseus
Serpula lacrymans	https://www.ncbi.nlm.nih.gov/genome/?term=Serpula+lacrymans
Sparassis crispa	https://www.ncbi.nlm.nih.gov/genome/?term=Sparassis+crispa
L. sulphureus ATCC52600	De Figueiredo *et.al* (1)

Table S11. The polysaccharide biosynthetic candidate genes in *L. sulphureus* NWAFU-1 genome.

Top Annotation	Gene ID	Species	E - Value	Ident (Per)	Accession Number
Beta-glucan synthesis-associated protein (GSAP)	g6340.t1	*Sparassis crispa*	0	68.51%	GBE78036.1
	g1889.t1	*Fomitopsis betulina*	0	76.31%	KAI0727283.1
	g7657.t1	*Lentinus tigrinus*	$6e^{-177}$	49.33%	RPD65389.1
	g6339.t1	*Sparassis crispa*	0	66.05%	GBE78036.1
	g11214.t1	*Sparassis crispa*	0	61.69%	GBE83229.1
GDP-mannose 4,6 dehydratase/GDP-Man dehydratase (GMD)	g11648.t1	*Sparassis crispa*	$9e^{-21}$	75.38%	GBE83607.1
	g7425.t1	*Wolfiporia cocos MD-104 SS10*	0	92.31%	PCH37529.1
	g13815.t1	*Armillaria ostoyae*	$7e^{-115}$	62.45%	SJL10393.1
	g11712.t1	*Diaporthe helianthi*	$2e^{-118}$	57.70%	POS69377.1
UDP-glucose 4-epimerase (UGE)	g6940.t1	*Polyporus arcularius HHB13444*	0	62.60%	TFK79498.1
	g6939.t1	*Fomitopsis betulina*	$6e^{-170}$	59.77%	KAI0732421.1
	g33.t1	*Wolfiporia cocos MD-104 SS10*	$5e^{-43}$	78.16%	PCH34309.1
Phosphomannose isomerase/Mannose-6-phosphate isomerase (PMI)	g7844.t1	*Wolfiporia cocos MD-104 SS10*	0	76.76%	PCH37027.1
	g9755.t2	*Wolfiporia cocos MD-104 SS10*	0	82.08%	PCH43549.1
Glucokinase (GK)	g4203.t1	*Daedalea quercina L-15889*	0	78.70%	KZT74519.1
	g5.t1	*Antrodia serialis*	0	73.46%	KAH9919555.1
Phosphoglucomutase/ Glucose phosphomutase (PGM)	g4792.t1	*Wolfiporia cocos MD-104 SS10*	0	90.99%	PCH36838.1
	g2633.t1	*Wolfiporia cocos MD-104 SS10*	0	76.90%	PCH38295.1
1,3-beta-glucan synthase (GLS)	g4749.t1	*Antrodia cinnamomea*	0	90.20%	KAI0943576.1
	g2718.t1	*Rhodofomes roseus*	0	85.89%	KAH9842776.1
Glucose-6-phosphate isomerase/ Phosphoglucose isomerase (PGI)	g6013.t1	*Wolfiporia cocos MD-104 SS10*	0	92.93%	PCH44991.1
Phosphofructokinase (FPK)	g13523.t1	*Wolfiporia cocos MD-104 SS10*	0	85.18%	PCH42160.1
UDP-Xyl synthase (UXS)	g3399.t1	*Moniliophthora roreri MCA 2997*	0	82.14%	ESK93589.1

Table S12. Terpenoid biosynthesis related enzymes in *L. sulphureus* NWAFU-1 genome.

Type	Entry	UniportKB database-based annotations	E-value	Indentify	Species	Accession Number
STS	g9694.t1	sesquiterpene synthase 14	2e-107	49.36%	*Postia placenta*	A0A348B788.1
	g11982.t1	sesquiterpene synthase 14	6e-139	54.46%	*Postia placenta*	A0A348B788.1
	g12368.t1	Sesquiterpene synthase Agr3	4e-143	72.28%	*Cyclocybe aegerita*	A0A5Q0QU70.1
	g3938.t1	Sesquiterpene synthase 2	0	81.10%	*Postia placenta*	A0A348B781.1
	g6730.t1	Terpene cyclase 25	8e-84	44.01%	*Postia placenta*	A0A348B781.1
	g6861.t1	Terpene cyclase 29	1e-46	51.97%	*Postia placenta*	A0A348B781.1
	g3034.t1	Sesquiterpene synthase 10	7e-74	46.30%	*Postia placenta*	B8PD44.1
	g10229.t1	Sesquiterpene synthase 14	2e-132	65.07%	*Postia placenta*	A0A348B781.1
	g10232.t1	Sesquiterpene synthase 14	2e-105	63.13%	*Postia placenta*	A0A348B781.1
	g13618.t1	Sesquiterpene synthase 10	4e-147	61.37%	*Postia placenta*	B8PD44.1
	g13621.t1	Sesquiterpene synthase 10	0	75.44%	*Postia placenta*	B8PD44.1
	g13710.t1	Sesquiterpene synthase 3	0	81.76%	*Postia placenta*	A0A348B781.1
	g571.t1	Sesquiterpene synthase 3	1e-160	62.24%	*Postia placenta*	A0A348B781.1
	g582.t1	Sesquiterpene synthase 3	1e-165	66.18%	*Postia placenta*	A0A348B781.1
	g581.t1	Sesquiterpene synthase 3	2e-73	63.10%	*Postia placenta*	A0A348B781.1
Mono-TS	g6731.t1	Monoterpene synthase 25	1e-151	67.00%	*Postia placenta*	A0A348B781.1
	g11971.t1	Monoterpene synthase 25	3e-44	42.68%	*Postia placenta*	A0A348B781.1
	g11978.t1	Monoterpene synthase 25	3e-64	44.39%	*Postia placenta*	A0A348B781.1
	g6728.t1	Monoterpene synthase 25	2e-80	41.39%	*Postia placenta*	A0A348B781.1
	g6862.t1	Monoterpene synthase 25	2e-108	53.87%	*Postia placenta*	A0A348B781.1
	g9796.t1	Monoterpene synthase 25	5e-125	60.20%	*Postia placenta*	A0A348B781.1
	g6885.t1	Monoterpene synthase 25	1e-132	60.00%	*Postia placenta*	A0A348B781.1
	g6859.t1	Monoterpene synthase 25	6e-136	61.64%	*Postia placenta*	A0A348B781.1
SQS	g317.t1	squalene synthase	0	74.73%	*Ganoderma lucidum*	A0SJQ5.1
PSY	g11613.t1	NADH dehydrogenase (ubiquinone) complex I, assembly factor 6	4e-48	37.02%	*Rattus norvegicus*	D3ZN43.1

Table S13. Identified sesquiterpenes derived from Basidiomycetes used to construct sesquiterpene phylogenetic tree.

STS entry	Accession No	Species	Reference
Cop1	XP_001832573		
Cop2	XP_001836556		
Cop3	XP_001832925	*Coprinus cinereus*	(2)
Cop4	XP_001836356		
Cop5	XP_001834007		
Cop6	XP_001832549		
Omp1-Omp10		*Omphalotus olearius*	(3)
Agr1- Agr11	MN146024 - MN146034	*Agrocybe aegerita*	(4)
CpSTS1- CpSTS18	LC436345 - LC436362	*Stereum hirsutum*	(5)

Table S14. Identify matrix between 8 STSs from NWAFU-1 and STS25.

	g6731.t1	g11971.t1	g11978.t1	g6728.t1	g6862.t1	g9796.t1	g6885.t1	g6859.t1	STS25
g6731.t1	100.00	39.18	43.93	39.68	80.63	57.19	57.96	82.08	64.95
g11971.t1	39.18	100.00	93.82	24.79	36.88	32.34	38.12	35.98	37.78
g11978.t1	43.93	93.82	100.00	33.33	40.76	42.60	42.60	39.62	43.32
g6728.t1	39.68	24.79	33.33	100.00	38.57	33.16	37.54	39.93	39.51
g6862.t1	80.63	36.88	40.76	38.57	100.00	52.65	51.76	78.70	56.58
g9796.t1	57.19	32.34	42.60	33.16	52.65	100.00	86.59	52.94	54.52
g6885.t1	57.96	38.12	42.60	37.54	51.76	86.59	100.00	53.42	57.19
g6859.t1	82.08	35.98	39.62	39.93	78.70	52.94	53.42	100.00	59.21
STS25	64.95	37.78	43.32	39.51	56.58	54.52	57.19	59.21	100.00

Percent Identity Matrix was analyzed online by Clustal Omega (https://www.ebi.ac.uk/Tools/msa/clustalo/).

Table S15. Sequence homology analysis for 9 PKSs from the strain NWAFU-1.

Entry	UniportKB database-based annotations	E-value	Indentify	Species	Accession Number
g11207.t1	Highly reducing polyketide synthase Stpks1	$1e^{-64}$	43.46%	*Strobilurus tenacellus*	A0A384XH94.1
g4528.t1	Highly reducing polyketide synthase Stpks1	0	45.30%	*Strobilurus tenacellus*	A0A384XH94.1
g4538.t1	Highly reducing polyketide synthase Stpks1	0	47.85%	*Strobilurus tenacellus*	A0A384XH94.1
g10146.t1	Highly reducing polyketide synthase Stpks1	$8e^{-49}$	31.07%	*Strobilurus tenacellus*	A0A384XH94.1
g10149.t1	Narbonolide/10-deoxymethynolide synthase PikA1	$1e^{-18}$	29.29%	*Streptomyces venezuelae*	Q9ZGI5.1
g10934.t1	Highly reducing polyketide synthase 40 PKS40	$2e^{-73}$	37.91%	*Fusarium pseudograminearum*	K3VA96.1
g10956.t1	Phthiocerol synthesis polyketide synthase type I PpsA	$5e^{-76}$	66.20%	*Mycobacterium tuberculosis*	Q7TXM0.1
g13526.t1	Orsellinic acid synthase ArmB	0	48.52%	*Amylocystis lapponica*	A0A2H3CTK0.1
g10220.t1	Chalcone synthase StlA	$7e^{-45}$	34.33%	*Dictyostelium discoideum*	Q55E72.1

Table S16. Sequence homology analysis for 11 NRPS-likes from the strain NWAFU-1.

Entry	homology	E-value	Indentify	Species	Accession Number
g5863	L-2-aminoadipate reductase	0	52.41%	*Schizosaccharomyces pombe 972h-*	P40976.3
g3837	Adenylate-forming reductase Nps11	0	40.91%	*Serpula lacrymans var. lacrymans S7.9*	F8P9P5.2
g823	Adenylate-forming reductase Nps11	0	38.93%	*Serpula lacrymans var. lacrymans S7.9*	F8P9P5.2
g838	Adenylate-forming reductase Nps11	0	36.96%	*Serpula lacrymans var. lacrymans S7.9*	F8P9P5.2
g10195	Adenylate-forming reductase Nps10	0	47.91%	*Heterobasidion annosum*	A0A1B1ZGB5.1
g13738	Adenylate-forming reductase Nps10	0	46.12%	*Heterobasidion annosum*	A0A1B1ZGB5.1
g7044	Adenylate-forming reductase Nps10	$1e^{-93}$	30.70%	*Heterobasidion annosum*	A0A1B1ZGB5.1
g4524	Adenylate-forming reductase Nps10	0	48.45%	*Heterobasidion annosum*	A0A1B1ZGB5.1
g4530	Adenylate-forming reductase Nps10	$7e^{-49}$	38.80%	*Heterobasidion annosum*	A0A1B1ZGB5.1
g4534	Adenylate-forming reductase Nps10	0	45.35%	*Heterobasidion annosum*	A0A1B1ZGB5.1
g10219	Mycosubtilin synthase subunit A	$6e^{-70}$	27.43%	*Bacillus subtilis*	Q9R9J1.1

Table S17. 46 identified NRPS-likes used in cluster analysis with 11 NRPS-Likes.

Entry	Genbank Accession No	Fungal species
ATEG_03630	Q0CRQ4	*Aspergillus terreus*
StbB	A0A193PS46	*Stachybotrys bisbyi*
CicB	A0A1U8QW91	*Emericella nidulans*
Nps11	F8P9P5	*Serpula lacrymans var. lacrymans*
Nps9	F8P2C8	*Serpula lacrymans var. lacrymans*
NCU05000	Q7RW48	*Neurospora crassa*
Nps10	A0A1B1ZGB5	*Heterobasidion annosum*
ATRR	A0A1U8QWA2	*Emericella nidulans*
Nps3	F8P1W3	*Serpula lacrymans var. lacrymans*
InvA2	A0A0S1RUN4	*Paxillus involutus*
InvA1	A0A0S2E7Z1	*Paxillus involutus*
InvA5	A0A0S2E7W7	*Paxillus involutus*
GreA	I6NXV7	*Suillus grevillei*
CC1G_06235	A8NVB7	*Coprinopsis cinerea*
CC1G_03009	A8NS27	*Coprinopsis cinerea*
InvA3	A0A0S2E7V8	*Paxillus involutus*
ATEG_03629	Q0CRQ5	*Aspergillus terreus*
InvA4	A0A0S2E7X0	*Paxillus involutus*
InvA6	A0A0S2E7W3	*Paxillus involutus*
ATEG_03631	Q0CRQ3	*Aspergillus terreus*
ATEG_07661	Q0CF73	*Aspergillus terreus*
MelA	Q0CRX1	*Aspergillus terreus*
ATEG_07662	Q0CF72	*Aspergillus terreus*
LnaA	B8NTZ9	*Aspergillus flavus*
ATEG_07659	Q0CF75	*Aspergillus terreus*
ApvA	Q0CWD0	*Aspergillus terreus*
MicA	Q5B7T4	*Emericella nidulans*
LnbA	B8NWW5	*Aspergillus flavus*
StbA	A0A193PS74	*Stachybotrys bisbyi*
LnaD	B8NU02	*Aspergillus flavus*
ATEG_03635	Q0CRP9	*Aspergillus terreus*
LnaC	B8NU01	*Aspergillus flavus*
ATEG_07660	Q0CF74	*Aspergillus terreus*
LnbC	B8NWW3	*Aspergillus flavus*
BtyA	Q0CU19	*Aspergillus terreus*
StbC	A0A193PS58	*Stachybotrys bisbyi*
PngA	Q0CBN5	*Aspergillus terreus*
AtrA	Q0CT94	*Aspergillus terreus*
ATEG_07663	Q0CF71	*Aspergillus terreus*
LnaF1	B8NU03	*Aspergillus flavus*
LnaB1	B8NU00	*Aspergillus flavus*
ATEG_03636	Q0CRP8	*Aspergillus terreus*
LnaE	B8NTZ8	*Aspergillus flavus*
LnaB2	B8NWW2	*Aspergillus flavus*
LnaF2	B8NWW7	*Aspergillus flavus*
LnbE	B8NWW6	*Aspergillus flavus*

Table S18. Identification of cytochrome P450 genes in *L. sulphureus* NWAFU-1 genome.

Protein ID	Gene ID	aa length	Identify	E-Value	Species	Accession
>g10525.t1	g10525	528	58.40%	0	*Postia placenta*	F1SY96.1
>g13454.t1	g13454	603	46.23%	$1e^{-153}$	*Postia placenta*	F1SY77.1
>g10166.t1	g10166	434	40.42%	$4e^{-109}$	*Postia placenta*	F1SYH1.1
>g11954.t1	g11954	680	57.53%	0	*Postia placenta*	F1SYI1.1
>g11954.t2		667	55.58%	0		
>g6382.t1	g6382	469	39.57%	$1e^{-109}$	*Coprinopsis cinerea*	A8NCK6.1
>g2541.t1	g2541	610	30.75%	$4e^{-53}$	*Hypoxylon pulicicidum*	A0A2I6PJ08.1
>g2541.t2		618	30.79%	$1e^{-48}$		
>g6907.t1	g6907	603	61.00%	0	*Postia placenta*	F1SY71.1
>g10839.t1	g10839	472	46.23%	$1e^{-153}$	*Postia placenta*	F1SY77.1
>g6884.t1	g6884	535	58.81%	0	*Postia placenta*	F1SY71.1
>g13485.t1	g13485	963	44.18%	$1e^{-137}$	*Postia placenta*	F1SYD1.1
>g13485.t2		995	41.94%	$6e^{-134}$		
>g5024.t1	g5024	505	41.18%	$4e^{-131}$	*Postia placenta*	F1SYD1.1
>g6909.t1	g6909	499	60.72%	0	*Postia placenta*	F1SY71.1
>g5477.t1	g5477	505	40.97%	$7e^{-125}$	*Postia placenta*	F1SYD1.1
>g2490.t1	g2490	525	44.57%	$3e^{-120}$	*Postia placenta*	F1SY96.1
>g10524.t1	g10524	266	64.31%	$5e^{-123}$	*Postia placenta*	F1SY96.1
>g5165.t1	g5165	387	42.50%	$4e^{-98}$	*Postia placenta*	F1SY74.1
>g11423.t1	g11423	574	42.89%	$3e^{-85}$	*Postia placenta*	F1SYH0.1
>g11423.t2		539	42.89%	$1e^{-85}$		
>g6947.t1	g6947	509	64.55%	0	*Postia placenta*	F1SY95.1
>g6944.t1	g6944	573	68.71%	0	*Postia placenta*	F1SY95.1<br>F1SY95.1
>g6944.t2		567	67.44%	0		
>g1210.t1	g1210	87	53.01%	$1e^{-22}$	*Postia placenta*	F1SYF1.1
>g5018.t1	g5018	542	38.29%	$1e^{-112}$	*Postia placenta*	F1SYD1.1
>g8113.t1	g8113	502	29.46%	$1e^{-31}$	*Postia placenta*	F1SY71.1
>g6523.t1	g6523	235	40.36%	$4e^{-28}$	*Hypoxylon pulicicidum*	A0A2I6PJ08.1
>g6978.t1	g6978	213	83.73%	$2e^{-128}$	*Postia placenta*	F1SYD1.1
>g6906.t1	g6906	481	55.13%	0	*Postia placenta*	F1SY71.1
>g10086.t1	g10086	461	38.17%	$5e^{-113}$	*Postia placenta*	F1SY91.1
>g6946.t1	g6946	505	61.48%	0	*Postia placenta*	F1SY95.1
>g10843.t1	g10843	434	39.89%	$7e^{-92}$	*Postia placenta*	F1SYH1.1
>g5478.t1	g5478	497	44.88%	$3e^{-148}$	*Postia placenta*	F1SYD1.1
>g3507.t1	g3507.	214	37.20%	$6e^{-39}$	*Postia placenta*	F1SY85.1
>g2488.t1	g2488	499	44.35%	$1e^{-141}$	*Postia placenta*	F1SY52.1
>g12143.t1	g12143	391	53.68%	$5e^{-66}$	*Postia placenta*	F1SYD1.1
>g2489.t1	g2489	466	44.62%	$6e^{-143}$	*Postia placenta*	F1SY96.1
>g9051.t1	g9051	361	32.48%	$1e^{-43}$	*Armillaria gallica*	A0A2H3CSA7.1
>g9051.t2		341	31.09%	$4e^{-42}$		A0A2H3CZX2.1
>g294.t1	g294	333	54.95%	$2e^{-125}$	*Postia placenta*	F1SYH4.1
>g10104.t1	g10104	435	45.16%	$5e^{-36}$	*Postia placenta*	F1SYF1.1
>g13495.t1	g13495	542	36.83%	$9e^{-107}$	*Postia placenta*	F1SY77.1

>g13495.t2	g13495	519	37.76%	$2e^{-103}$	*Postia placenta*	F1SY77.1
>g5022.t1	g5022	476	42.20%	$3e^{-138}$	*Postia placenta*	F1SYD1.1
>g10103.t1	g10103	469	37.50%	$2e^{-61}$	*Postia placenta*	F1SY68.1
>g5258.t1	g5258	512	36.47%	$2e^{-84}$	*Postia placenta*	F1SYD1.1
>g5258.t2	g5258	535	36.47%	$7e^{-84}$	*Postia placenta*	F1SYD1.1
>g13502.t1	g13502	517	35.04%	$6e^{-98}$	*Postia placenta*	F1SYI1.1
>g10194.t1	g10194	511	28.70%	$5e^{-38}$	*Postia placenta*	F1SY73.1
>g10194.t2		496	30.14%	$1e^{-34}$		F1SY96.1
>g9077.t1	g9077	400	51.70%	$7e^{-43}$	*Postia placenta*	F1SY68.1
>g9075.t1	g9075	364	33.45%	$4e^{-62}$	*Postia placenta*	F1SY68.1
>g3209.t1	g3209	1045	56.25%	0	*Postia placenta*	F1SYH1.1
>g5483.t1	g5483	1184	41.09%	$5e^{-119}$	*Postia placenta*	F1SYD1.1
>g5483.t2		1209	41.09%	$7e^{-119}$		
>g5483.t3		1207	41.09%	$6e^{-119}$		
>g9054.t1	g9054	539	30.30%	$2e^{-69}$	*Trichoderma virens*	G9N4A8.1
>g13530.t1	g13530	544	31.04%	$3e^{-71}$	*Trichoderma virens*	G9N4A8.1
>g4668.t1	g4668	494	35.46%	$8e^{-91}$	*Postia placenta*	F1SYF1.1
>g13378.t1	g13378	559	54.79%	0	*Postia placenta*	F1SYI9.1
>g9046.t1	g9046	509	35.62%	$2e^{-92}$	*Armillaria gallica*	A0A2H3CZX2.1
>g1155.t1	g1155	544	72.61%	0	*Postia placenta*	F1SYH0.1
>g751.t1	g751	562	55.76%	0	*Postia placenta*	F1SY74.1
>g8635.t1	g8635	594	55.27%	0	*Armillaria gallica*	A0A2H3CZX2.1
>g1231.t1	g1231	551	68.00%	0	*Postia placenta*	F1SYH0.1
>g632.t1	g632	565	57.52%	0	*Postia placenta*	F1SYI9.1
>g12550.t1	g12550	1437	61.27%	0	*Postia placenta*	F1SYI9.1
>g8402.t1	g8402	548	53.44%	0	*Ustilago maydis*	P49602.1
>g3509.t1	g3509	543	30.71%	$1e^{-74}$	*Postia placenta*	F1SYH0.1
>g1230.t1	g1230	726	68.95%	0	*Postia placenta*	F1SYH0.1
>g4797.t1	g4797	547	30.04%	$2e^{-65}$	*Fusarium pseudograminearum*	P0DPA4.1
>g9019.t1	g9019	572	36.40%	$1e^{-90}$	*Armillaria gallica*	A0A2H3CZX2.1
>g12483.t1	g12483	605	63.89%	0	*Postia placenta*	F1SY74.1
>g13527.t1	g13527	519	28.09%	$3e^{-61}$	*Trichoderma virens*	G9N4A8.1
>g12484.t1	g12484	605	62.23%	0	*Postia placenta*	F1SY74.1
>g4794.t1	g4794	565	31.08%	$2e^{-64}$	*Uromyces viciae-fabae*	O00061.1
>g2540.t1	g2540	462	32.37%	$9e^{-70}$	*Hypoxylon pulicicidum*	A0A2I6PJ08.1
>g11455.t1	g11455	536	54.00%	0	*Postia placenta*	F1SYI1.1
>g6524.t1	g6524	566	32.32%	$2e^{-56}$	*Fusarium pseudograminearum*	P0DPA4.1
>g4796.t1	g4796	562	30.60%	$2e^{-62}$	*Uromyces viciae-fabae*	O00061.1
>g4166.t1	g4166	514	43.71%	$8e^{-146}$	*Postia placenta*	F1SY91.1
>g703.t1	g703	545	49.81%	$2e^{-178}$	*Postia placenta*	F1SY85.1
>g1309.t1	g1309	512	59.43%	0	*Postia placenta*	F1SY96.1
>g11955.t1	g11955	599	57.14%	0	*Postia placenta*	F1SYI1.1
>g3343.t1	g3343	561	58.24%	0	*Postia placenta*	F1SY70.1
>g9792.t1	g9792	329	39.77%	$8e^{-32}$	*Postia placenta*	F1SYF1.1

>g729.t1	g729	507	41.09%	8e^{-129}	*Postia placenta*	F1SYH1.1
>g10234.t1	g10234	498	58.65%	2e^{-169}	*Postia placenta*	F1SY74.1
>g10176.t1	g10176	510	39.79%	1e^{-116}	*Postia placenta*	F1SYH1.1
>g668.t1	g668	553	43.89%	9e^{-151}	*Ustilago maydis*	P49602.1
>g13251.t1	g13251	553	43.69%	2e^{-150}	*Ustilago maydis*	P49602.1
>g3015.t1	g3015	555	55.77%	0	*Postia placenta*	F1SY96.1
>g12032.t1	g12032	588	37.34%	8e^{-121}	*Postia placenta*	F1SY74.1
>g11674.t1	g11674	511	42.31%	6e^{-127}	*Postia placenta*	F1SYH1.1
>g3529.t1	g3529	493	29.67%	9e^{-68}	*Postia placenta*	F1SYB6.1
>g4795.t1	g4795	544	31.75%	2e^{-65}	*Fusarium pseudograminearum*	P0DPA4.1
>g808.t1	g808	483	49.34%	2e^{-163}	*Postia placenta*	F1SYH1.1
>g5393.t1	g5393	501	60.74%	0	*Postia placenta*	F1SY85.1
>g10575.t1	g10575	528	58.75%	0	*Postia placenta*	F1SY96.1
>g11488.t1	g11488	569	34.79%	2e^{-98}	*Postia placenta*	F1SYB6.1
>g780.t1	g780	470	42.98%	1e^{-112}	*Postia placenta*	F1SYH1.1
>g10579.t1	g10579	528	59.20%	0	*Postia placenta*	F1SY96.1
>g245.t1	g245	317	32.27%	1e^{-30}	*Postia placenta*	F1SYH0.1
>g7482.t1	g7482	408	34.92%	4e^{-65}	*Postia placenta*	F1SYH1.1
>g4587.t1	g4587	315	28.10%	4e^{-30}	*Postia placenta*	F1SYG6.1
>g2961.t1	g2961	374	39.48%	3e^{-59}	*Postia placenta*	F1SY68.1
>g10191.t1	g10191	405	36.10%	3e^{-67}	*Postia placenta*	F1SYH1.1
>g1398.t1	g1398	375	43.55%	1e^{-25}	*Postia placenta*	F1SYB6.1
>g4590.t1	g4590	852	27.35%	1e^{-26}	*Postia placenta*	F1SYG6.1
>g10116.t1	g10116	180	46.31%	6e^{-45}	*Postia placenta*	F1SYF1.1
>g8401.t1		375	42.29%	2e^{-66}		
>g8401.t2	g8401	377	42.69%	1e^{-68}	*Postia placenta*	F1SY68.1
>g8401.t3		368	42.25%	1e^{-69}		
>g10125.t1	g10125	165	45.64%	3e^{-43}	*Postia placenta*	F1SY68.1
>g10254.t1	g10254	280	42.08%	1e^{-39}	*Postia placenta*	F1SY68.1
>g10123.t1	g10123	197	40.13%	5e^{-38}	*Postia placenta*	F1SY68.1
>g1192.t1	g1192	177	51.91%	9e^{-41}	*Postia placenta*	F1SYF1.1
>g3234.t1	g3234	189	44.53%	2e^{-39}	*Postia placenta*	F1SYF1.1
>g10124.t1	g10124	206	46.43%	2e^{-39}	*Postia placenta*	F1SYF1.1
>g10237.t1	g10237	276	40.83%	2e^{-34}	*Postia placenta*	F1SY68.1
>g1211.t1	g1211	177	51.15%	8e^{-40}	*Postia placenta*	F1SYF1.1
>g7402.t1	g7402	335	32.81%	8e^{-25}	*Fusarium pseudograminearum*	P0DPA4.1
>g10102.t1	g10102	205	42.86%	6e^{-38}	*Postia placenta*	F1SY68.1
>g10238.t1	g10238	231	40.61%	4e^{-39}	*Postia placenta*	F1SY68.1
>g10120.t1	g10120	188	41.67%	1e^{-47}	*Postia placenta*	F1SY68.1
>g10108.t1	g10108	170	50.00%	9e^{-41}	*Postia placenta*	F1SYF1.1
>g10106.t1	g10106	245	46.27%	3e^{-39}	*Postia placenta*	F1SYF1.1
>g10106.t2		228	46.27%	2e^{-39}		
>g2156.t1	g2156	188	38.73%	2e^{-25}	*Postia placenta*	F1SY68.1
>g839.t1	g839	223	45.07%	4e^{-40}	*Postia placenta*	F1SY68.1
>g10527.t1	g10527	196	39.18%	3e^{-29}	*Postia placenta*	F1SY96.1
>g10168.t1	g10168	383	35.23%	1e^{-56}	*Postia placenta*	F1SYH1.1

>g10118.t1	g10118	228	46.21%	$3e^{-34}$	*Postia placenta*	F1SY68.1
>g10119.t1	g10119	152	44.36%	$4e^{-38}$	*Postia placenta*	F1SY68.1
>g11485.t1	g11485	210	41.07%	$7e^{-33}$	*Postia placenta*	F1SY68.1
>g1334.t1	g1334	281	43.61%	$2e^{-32}$	*Postia placenta*	F1SYF1.1
>g3235.t1	g3235	145	47.13%	$4e^{-24}$	*Postia placenta*	F1SYC2.1
>g9477.t1	g9477	211	51.45%	$2e^{-37}$	*Postia placenta*	F1SYF1.1
>g2143.t1	g2143	344	30.86%	$2e^{-34}$	*Postia placenta*	F1SYF1.1
>g2143.t2		353	30.86%	$2e^{-34}$		
>g10523.t1	g10523	189	55.42%	$5e^{-61}$	*Postia placenta*	F1SY96.1
>g3041.t1	g3041	200	38.00%	$2e^{-22}$	*Postia placenta*	F1SY68.1
>g10107.t1	g10107	132	49.04%	$3e^{-30}$	*Postia placenta*	F1SY68.1
>g5380.t1	g5380	201	60.87%	$5e^{-19}$	*Postia placenta*	F1SY96.1
>g5380.t2		205	54.12%	$3e^{-19}$		F1SY85.1
>g5380.t3		205	57.53%	$5e^{-17}$		F1SY85.1

Table S19. The NMR data of three compounds from the strain NWAFU-1.

Compounds	^{1}H NMR δ	^{13}C NMR δ	Reference
**Ergosta-4,6,8(14),22-tetraen-3-one (1)**	(400 MHz, $CDCl_3$) 6.60 (1H), 6.03 (1H), 5.73 (1H), 5.23(1H), 2.51 (1H), 2.40 (1H), 2.15 (1H), 2.05 (1H), 1.83 (1H), 1.70 (1H), 1.59 (1H), 1.48 (1H), 1.27 (1H), 1.06 (1H), 0.98 (3H), 0.94 (1H), 0.84 (3H).	(100 MHz, $CDCl_3$) 199.66, 164.53, 156.24, 135.15, 134.16, 132.68, 124.61, 124.57, 123.15, 55.85, 44.48, 44.14, 43.02, 39.44, 36.91, 35.74, 34.28, 33.24, 27.87, 25.52, 21.37, 20.14, 19.81, 19.13, 19.10, 17.79, 16.80.	Xu, *et al* (6).
**3β-hydroxy-5,9-epoxy-(22*E*,24*R*)-ergosta-7,22-dien-6-one (4)**	(500 MHz, CD_3OD) 5.48 (1H), 5.14 (2H), 3.84 (1H), 3.20 (3H), 2.64 (1H), 2.20 (1H), 1.92 (3H), 1.78 (3H), 1.64 (1H), 1.50 (2H), 1.38 (3H), 1.20 (6H), 0.94 (3H), 0.90 (2H), 0.82 (2H), 0.78 (6H), 0.58 (3H).	(125 MHz, CD_3OD) 200.14, 165.03, 136.63, 133.64, 120.92, 80.21, 76.17, 67.81, 57.48, 52.83, 46.22, 44.33, 42.81, 41.60, 37.17, 36.17, 34.36, 30.99, 29.36, 29.04, 26.62, 23.40, 21.59, 20.57, 20.44, 20.08, 18.16, 12.60.	Yu, *et al* (7).
**Dehydrosulphurenic acid (5)**	(400 MHz, CD_3OD) 5.91 (1H), 5.33 (1H), 4.25 (1H), 3.33 (2H), 3.16 (1H), 2.20 (7H), 2.00 (7H), 1.80 (4H), 1.60 (5H), 1.42 (2H), 1.30 (2H), 1.10 (1H), 1.08 (1H), 1.06 (6H), 1.00 (3H), 0.98 (6H), 0.92 (3H), 0.88 (3H), 0.80 (1H), 0.68 (3H).	(100 MHz, CD_3OD) 180.17, 156.53, 147.71, 142.21, 122.86, 116.91, 107.51, 79.58, 74.89, 52.91, 51.86, 46.84, 45.33, 39.76, 39.20, 38.64, 37.31, 37.17, 35.04, 33.04, 32.39, 28.81, 28.47, 24.03, 23.30, 22.32, 22.20, 17.97, 16.70, 16.48.	Chepkirui, *et al* (8).
**Sulfurenic acid (11)**	(400 MHz, DMSO-d_6) 4.72 (1H), 4.63 (1H), 4.26 (2H), 4.00 (1H), 3.20 (1H), 3.00 (1H), 2.12 (4H), 2.00 (3H), 1.80 (4H), 1.75 (2H), 1.60 (5H), 1.42 (9H), 1.10 (2H), 0.90 (7H), 0.85 (6H), 0.80 (3H), 0.70 (6H).	(100 MHz, DMSO-d_6) 177.14, 154.76, 133.96, 133.89, 106.83, 76.76, 71.01, 50.81, 50.00, 47.34, 45.33, 44.11, 38.51, 37.86, 36.63, 35.24, 33.24, 31.45, 30.64, 29.05, 28.12, 27.58, 26.52, 21.62, 21.54, 20.12, 18.91, 17.90, 17.47, 16.02, 15.86.	Yoshikawa, *et al* (9).

Table S20. The candidate genes related to Tetracyclic triterpene derivatives synthesis in *L. sulphureus* NWAFU-1 genome.

Function	Gene ID	Annotation				
		homology	Species	Identities	E -Value	Accession Number
squalene synthase (SQS)	g317	ERG9_GANLU<br>FDFT_USTMA	*Ganoderma lucidum*<br>*Ustilago maydis*	74.70%<br>46.20%	0<br>$4.3e^{-146}$	A0SJQ5<br>Q92459
squalene epoxidase (SE)	g5409	ERG1_GANLU	*Ganoderma lucidum*	62.30%	$2e^{-085}$	KY211742
lanosterol synthase (OSC)	g10772	ERG7_GANLU	*Ganoderma lucidum*	70.00%	0	ADD60469.1
C14-demethylase	g8402<br>g668<br>g13251	CP51_USTMA	*Ustilago maydis*	53.00%<br>44.00%<br>43.69%	0<br>$9e^{-151}$<br>$2e^{-150}$	P49602.1
C14-reductase	g11705	PiERG24	*Phellinus igniarius*	70.00%	0	QXU63178
C24-methylase	g3744	C8Q71DRAFT_775078	*Rhodofomes roseus*	80.00%	0	KAH9833332
C21-oxidatase	g11955<br>g11954	CYP5150L8	*Ganoderma lucidum*	48.00%<br>47.00%	e^{-143}<br>e^{-142}	AGL94943.1
C7-α-hydroxylase	g668	CYP7A1<br>CYP7B1	*Homo sapiens*<br>*Rattus norvegicus*	20.00%<br>21.00%	e^{-013}<br>$5e^{-017}$	NM_000780<br>NP_062011
C7-αOH-glycosyltransferase	g7567<br>g557<br>g519	UGT74AE2	*Panax ginseng*	29.00%<br>28.00%<br>28.00%	$5e^{-13}$<br>e^{-12}<br>$2e^{-12}$	A0A0A6ZFR4

Laetiporus sulphureus var. miniatus strain NAAS04758 18S ribosomal RNA gene, partial sequence; internal transcribed spacer 1, 5.8S ribosomal RNA gene, and internal transcribed spacer 2, complete sequence; and 28S ribosomal RNA gene, partial sequence

Sequence ID: KP004986.1 Length: 584 Number of Matches: 1

Range 1: 1 to 584 GenBank Graphics   ▼ Next Match ▲ Previous Match

Score	Expect	Identities	Gaps	Strand
1074 bits(581)	0.0	583/584(99%)	0/584(0%)	Plus/Plus

```
Query  17   TGCGGAAGGATCATTAACGAACAACGGGGCTCGCCCCTTGTCCACAAACACACCCCCGTG  76
            ||||||||||||||||||||||||||||||||||||||||||||||||||||||||||||
Sbjct  1    TGCGGAAGGATCATTAACGAACAACGGGGCTCGCCCCTTGTCCACAAACACACCCCCGTG  60

Query  77   CACGTCGAAGGCCCGGCTCGTTGAGTGGGTGGGCGACCGCCCAGGATTCGTAGCCTCGCT  136
            ||||||||||||||||||||||||||||||||||||||||||||||||||||||||||||
Sbjct  61   CACGTCGAAGGCCCGGCTCGTTGAGTGGGTGGGCGACCGCCCAGGATTCGTAGCCTCGCT  120

Query  137  TTCTTTACACAAACTTCGGAATGTAGATCGGAATGTCTATTGGGTATACAAATATAATAT  196
            ||||||||||||||||||||||||||||||||||||||||||||||||||||||||||||
Sbjct  121  TTCTTTACACAAACTTCGGAATGTAGATCGGAATGTCTATTGGGTATACAAATATAATAT  180

Query  197  AACTTTCAGCAACGGATCTCTTGGCTCTCGCATCGATGAAGAACGCAGCGAAATGCGATA  256
            ||||||||||||||||||||||||||||||||||||||||||||||||||||||||||||
Sbjct  181  AACTTTCAGCAACGGATCTCTTGGCTCTCGCATCGATGAAGAACGCAGCGAAATGCGATA  240

Query  257  AGTAATGTGAATTGCAGAATTCAGTGAATCATCGAATCTTTGAACGCACCTTGCGCTCCT  316
            ||||||||||||||||||||||||||||||||||||||||||||||||||||||||||||
Sbjct  241  AGTAATGTGAATTGCAGAATTCAGTGAATCATCGAATCTTTGAACGCACCTTGCGCTCCT  300

Query  317  TGGCATTCCGAGGAGCATGCCTGTTTGAGTGTCATGAAACCCTCAACCCCTGCCATCTTT  376
            ||||||||||||||||||||||||||||||||||||||||||||||||||||||||||||
Sbjct  301  TGGCATTCCGAGGAGCATGCCTGTTTGAGTGTCATGAAACCCTCAACCCCTGCCATCTTT  360

Query  377  GCGGATGAGCGTCGGTTGGATTTTGGAGGCTGCCGGACTCGTTCGGCTCCTCTTGAAAGC  436
            ||||||||||||||||||||||||||||||||||||||||||||||||||||||||||||
Sbjct  361  GCGGATGAGCGTCGGTTGGATTTTGGAGGCTGCCGGACTCGTTCGGCTCCTCTTGAAAGC  420

Query  437  ATAGTGAAGCTTGGACCTGACCGACCGGGTGGACGTGATAGAAAGTCACCGTCGACCGAA  496
            ||||||||||||||||||||||||||||||||||||||||||||||||||||||||||||
Sbjct  421  ATAGTGAAGCTTGGACCTGACCGACCGGGTGGACGTGATAGAAAGTCACCGTCGACCGAA  480

Query  497  GGGTCCGTCGCCGAACGGTTCAAGCTTTGTTTACCGTCTTCGGACGAACATCTCTGACCT  556
            |||||||||| |||||||||||||||||||||||||||||||||||||||||||||||||
Sbjct  481  GGGTCCGTCGCTGAACGGTTCAAGCTTTGTTTACCGTCTTCGGACGAACATCTCTGACCT  540

Query  557  CTGACCTCAAATCAGGTAGGACTACCCGCTGAACTTAAGCATAT  600
            ||||||||||||||||||||||||||||||||||||||||||||
Sbjct  541  CTGACCTCAAATCAGGTAGGACTACCCGCTGAACTTAAGCATAT  584
```

Figure S1. ITS alignment of the strain NWAFU-1.

The ITS of strain NWAFU-1 was aligned to *Laetiporus sulphureus* var. miniatus strain NAAS04758 (KP004986.1).

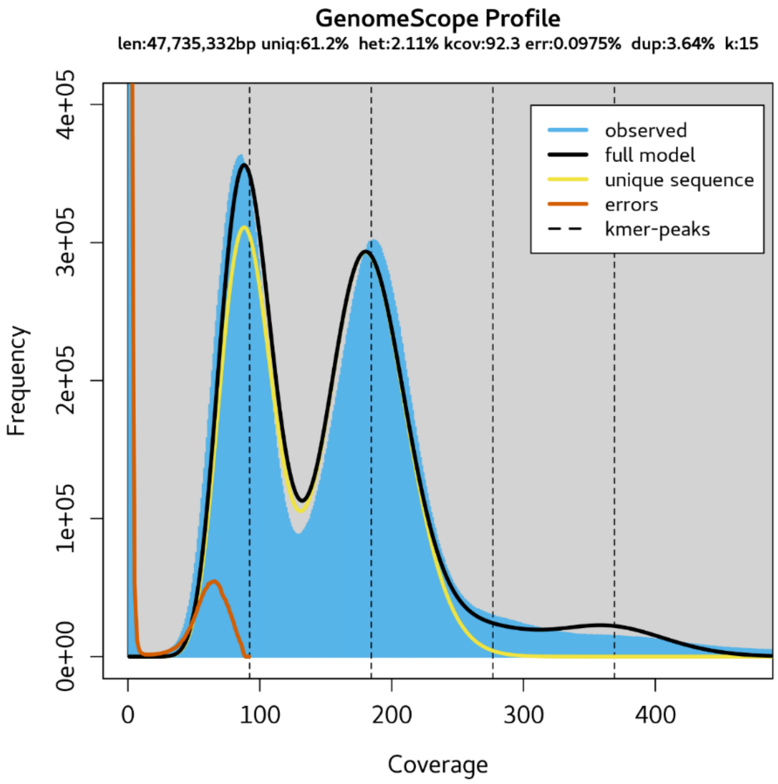

Figure S2. Kmer-Depth and Kmer Species-Frequency Distribution Plot.

The blue line represents the actual K-mer curve, the black line is the k-mer curve estimated by the model, the yellow line is the K-mer curve corresponding to the unique data, the red line represents the error curve due to sequencing errors, and the dashed line represents speculation K-mer peak.

Using the reads obtained by sequencing, K-mer-based analysis was used to estimate the genome size and heterozygosity. A K-mer refers to a sequence of K bp in length. Iteratively select a sequence of length K bases from a continuous sequence. If the length of the sequence is L and the length of the K-mer is K, then L-K+1 K-mers can be obtained. We take K-mers for the reads obtained by sequencing, and then count the frequency of each K-mer. According to the Lander_waterman algorithm, the genome size (G) satisfies the following formula:

$$C_{base} = C_{k-mer} \times \frac{L}{L-K+1}$$

$$G = \frac{n_{k-mer}}{C_{k-mer}} = \frac{n_{base}}{C_{base}}$$

C_{base} and $C_{k\text{-}mer}$ are the expected depth of coverage and K-mer, and n_{base} and $n_{k\text{-}mer}$ are the total number of bases and the total number of K-mers in the sequence. In the case of a certain amount of data, the depth frequency of K-mer is subject to Poisson distribution, so the peak of the K-mer depth frequency distribution is the corresponding depth, which is used as an estimate of the expected depth of K-mer.

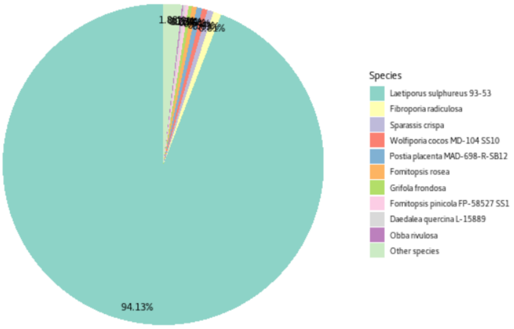

Figure S3. Species distribution map of Nr database alignment to sequences.

According to the results of the Nr library match, the top 10 species were counted and the rest were classified as other species, and the distribution of these species was mapped according to their proportion.

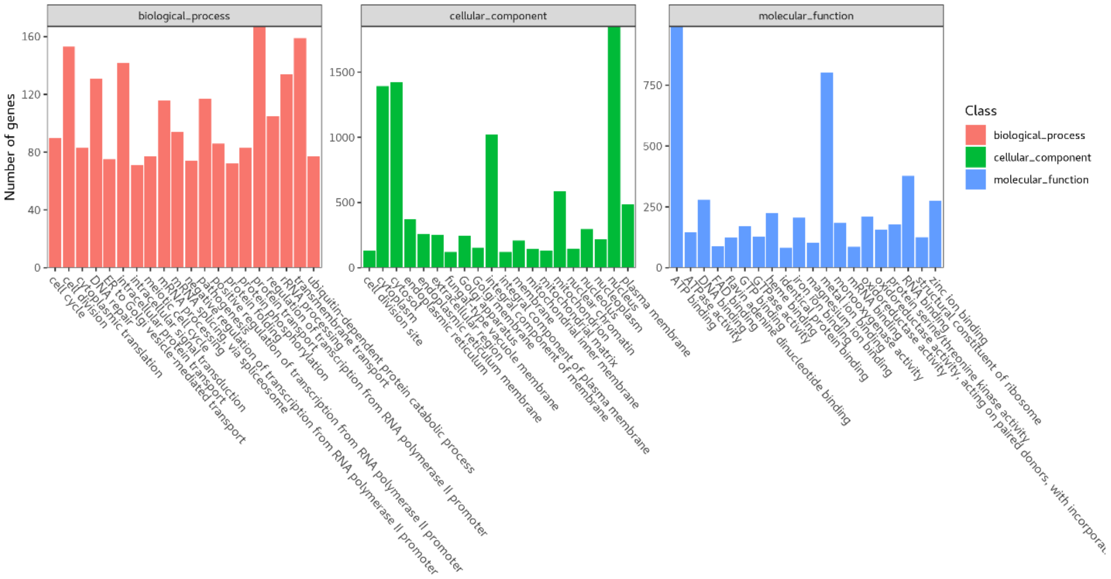

Figure S4. Statistical map of functional annotation classification based on GO database

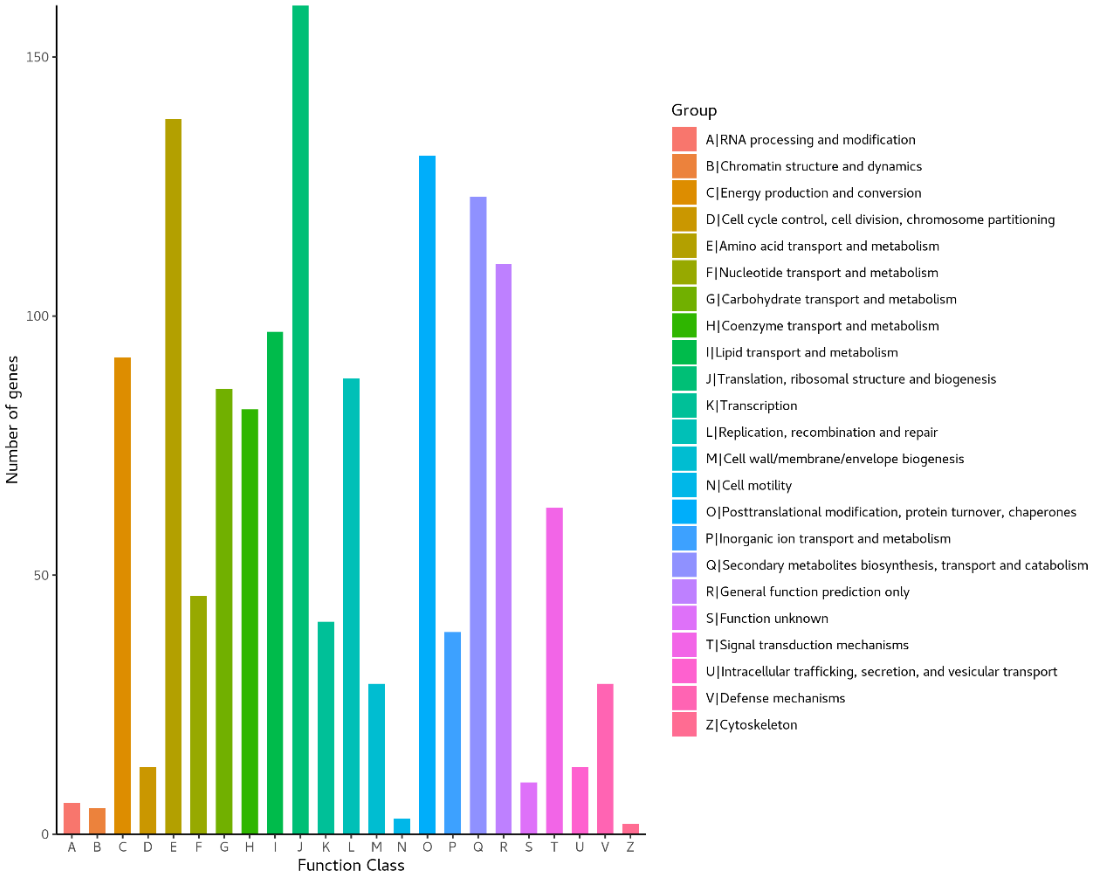

Figure S5. Statistical Chart of COG Functional Annotated Classification.

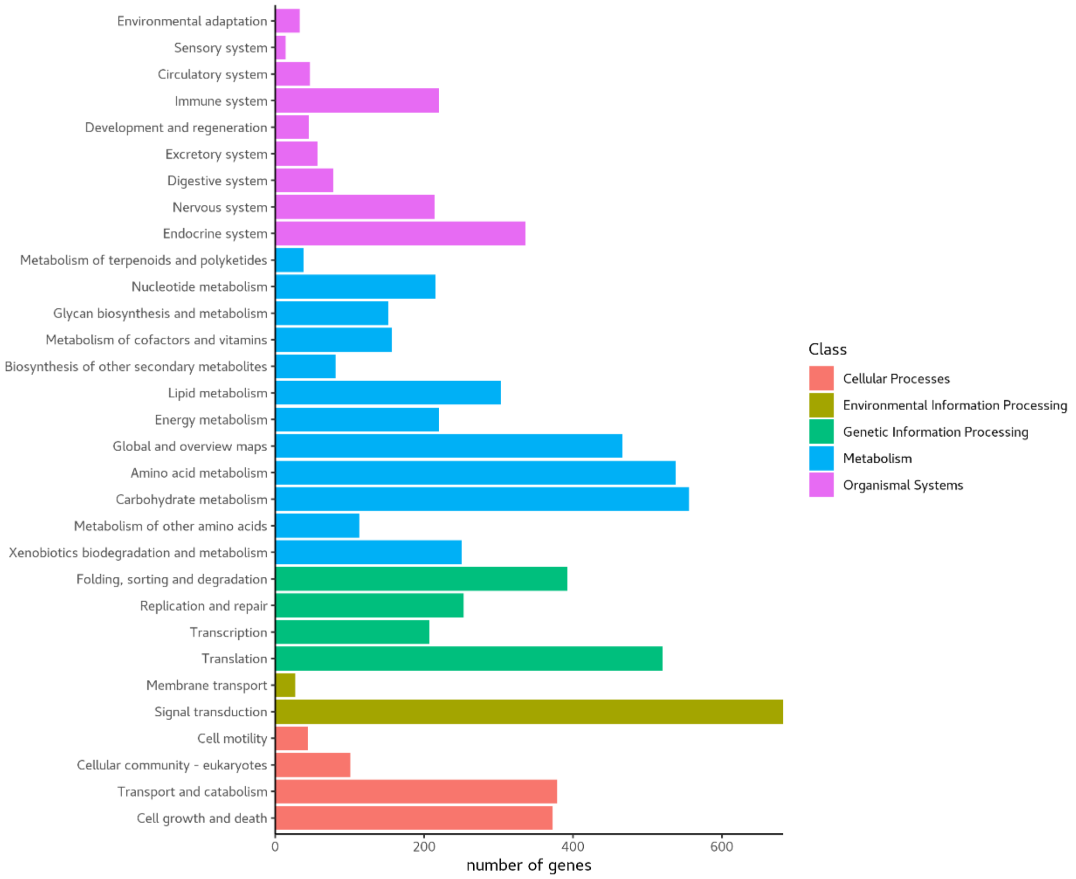

Figure S6. KEGG Pathway Functional Classification Chart.

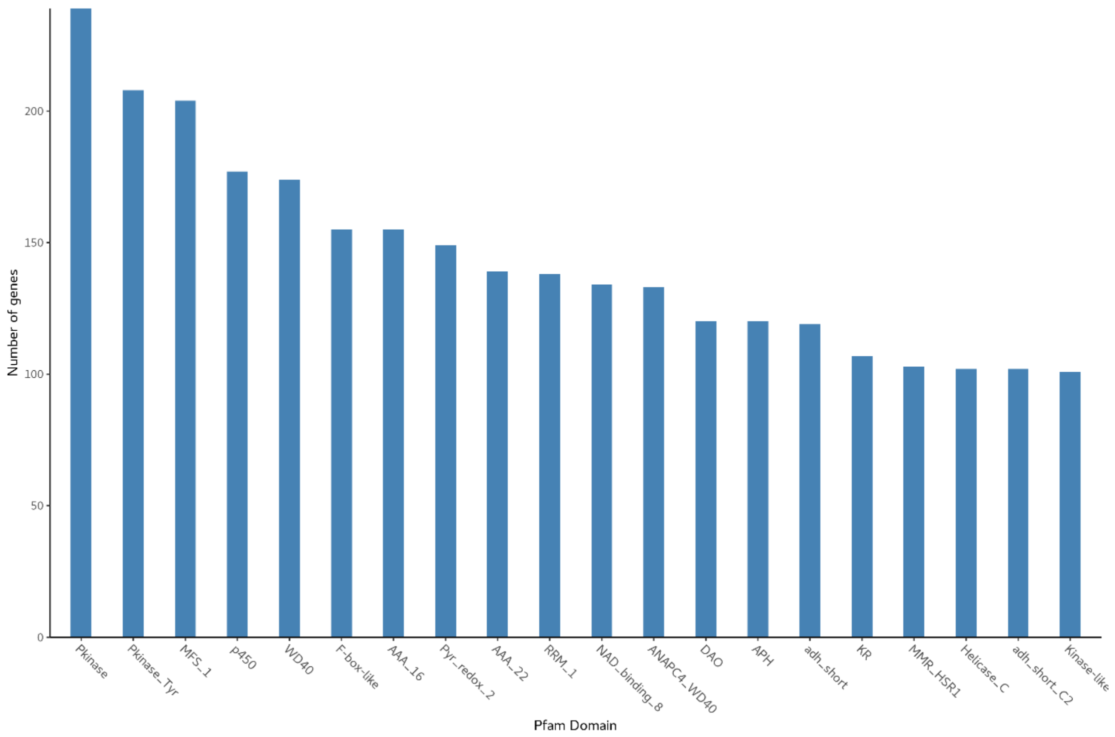

Figure S7. Domain annotation based on the Pfam database.

Statistical summaries of genes annotated for each domain are made and the top 20 most annotated domains are plotted.

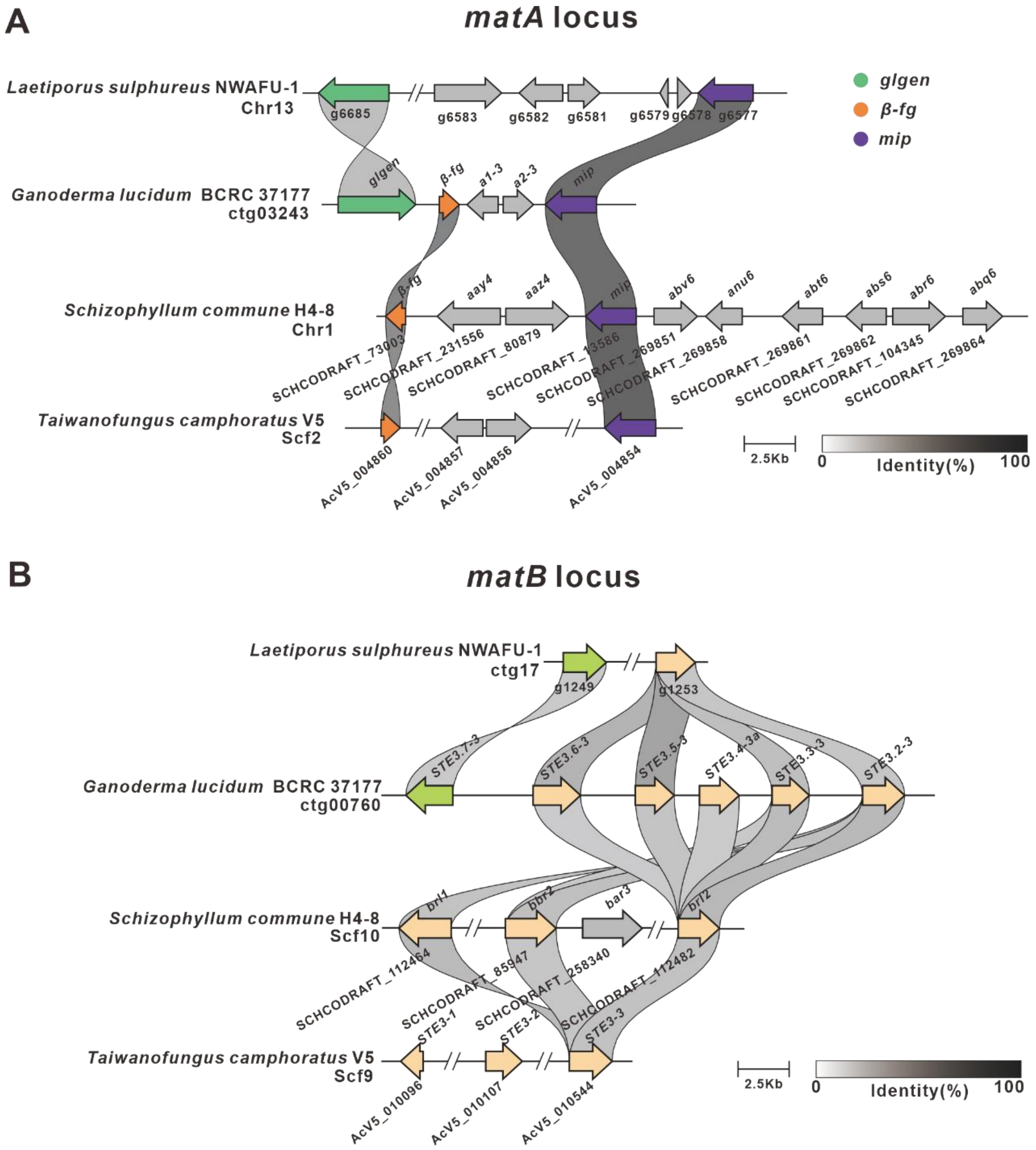

Figure S8. Comparison of the similarity of related genes on the matA and matB loci of NWAFU-1 and four mushroom species.

The loci used for comparison are from the reported literature(10-13), and the images were drawn by clinker (14).

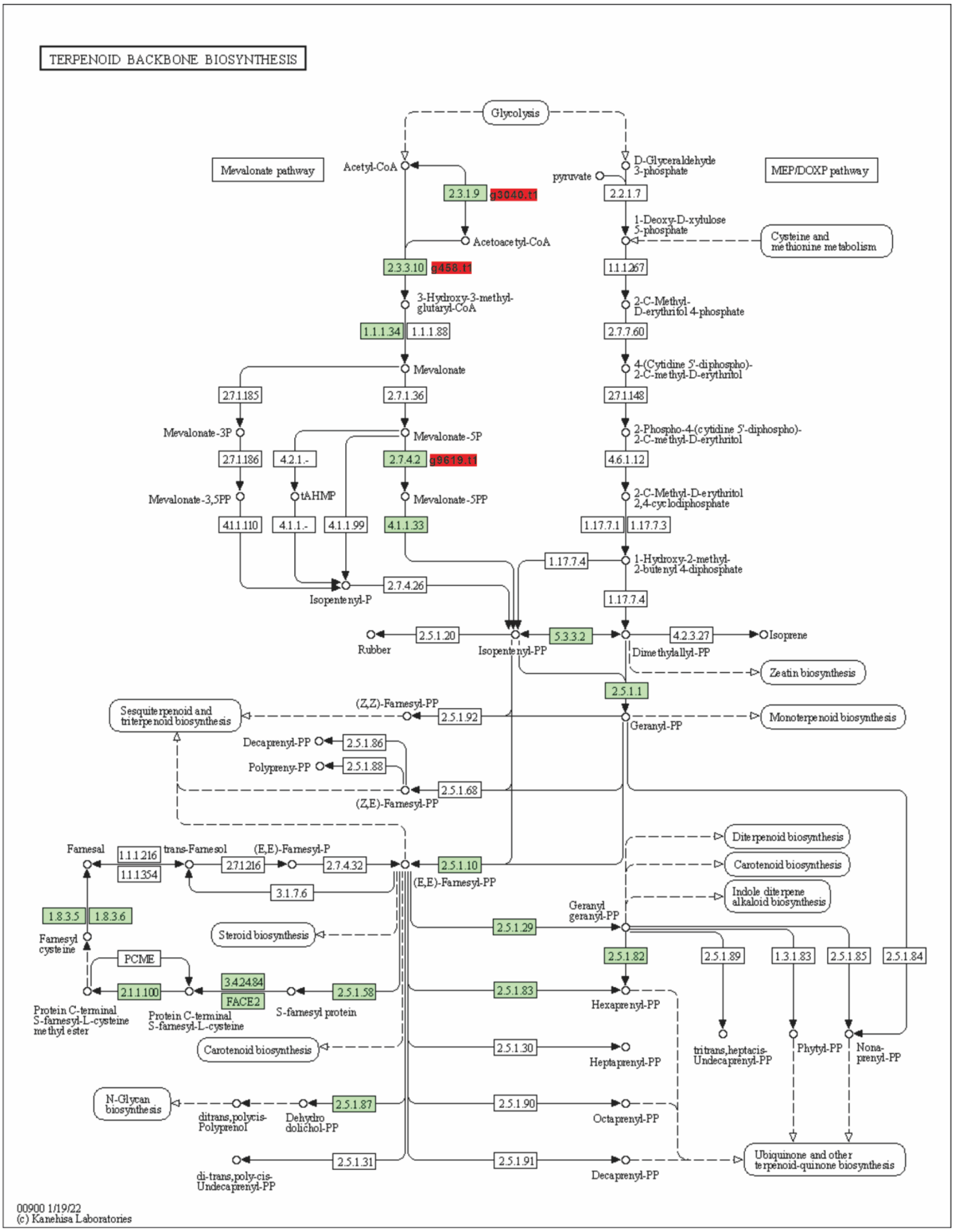

Figure S9. Annotation of the terpenoid backbone biosynthetic pathway of NWAFU-1 using KAAS.

The genes with green background represent NWAFU-1-derived genes annotated into the pathway, and proteins with red background represent specific NWAFU-1-derived proteins involved in MVP. KAAS annotation is done by KEGG Automatic Annotation Server Ver. 2.1.

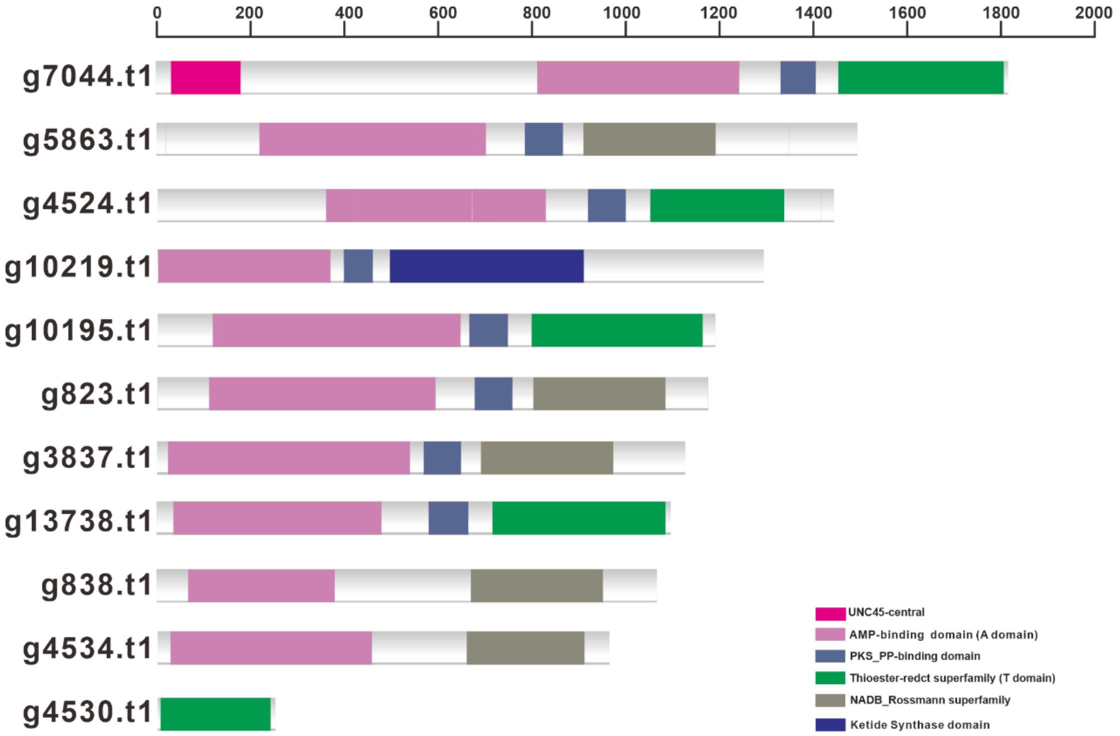

Figure S10. Domain analysis of 11 NRPS-like enzymes from the genome of the strain NWAFU-1.

Domain analysis was performed by CCD (https://www.ncbi.nlm.nih.gov/Structure/cdd/wrpsb.cgi).

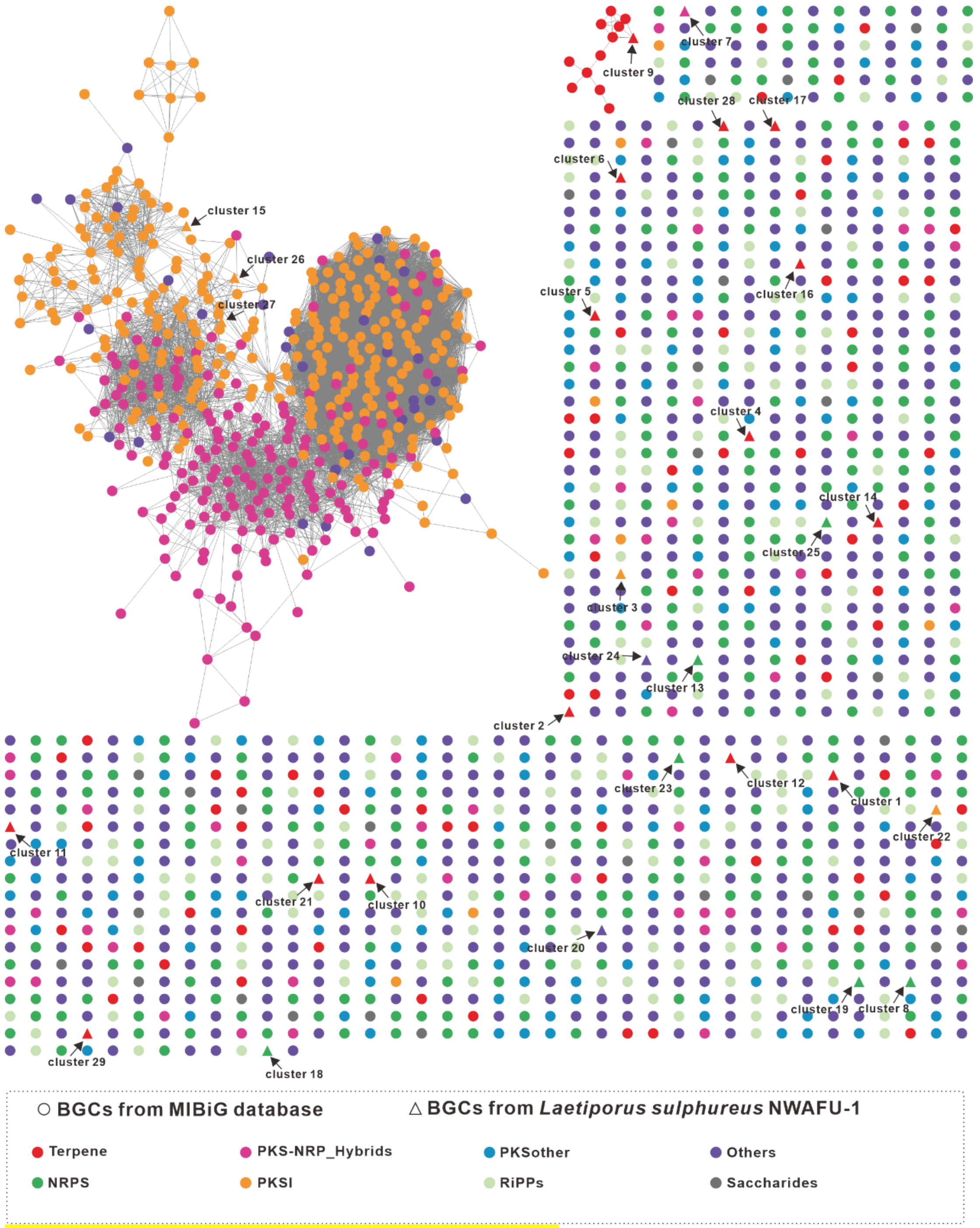

Figure S11. GCF analysis of BGCs from the strain NWAFU-1.

GCF similarity network of the BGCs was obtained using BiG-SCAPE (15). Each node represents a BGC and those with similar Pfam domain metrics are connected by edges. A cut-off of 0.80 was used for the analysis and the final similarity network was visualized using Cytoscape 3.9.1.

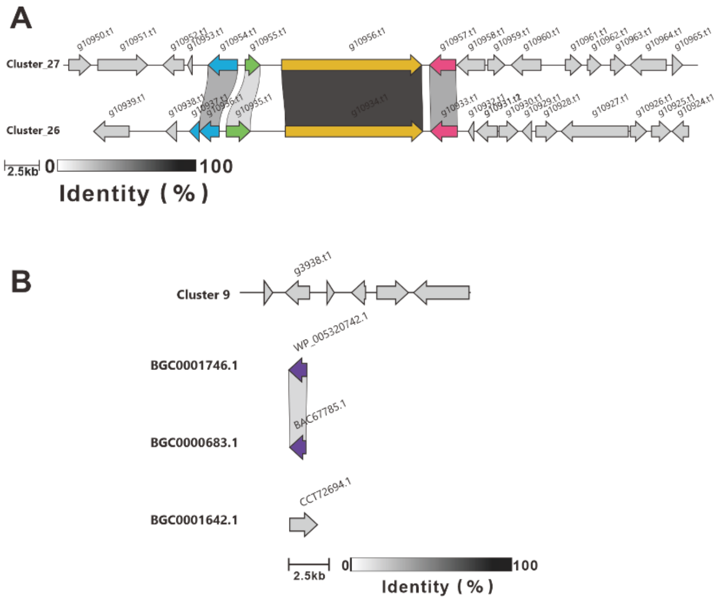

Figure S12. BGC similarity analysis of clusters 26 and 27(A), and 9 and others (B). The images were drawn by clinker (14).

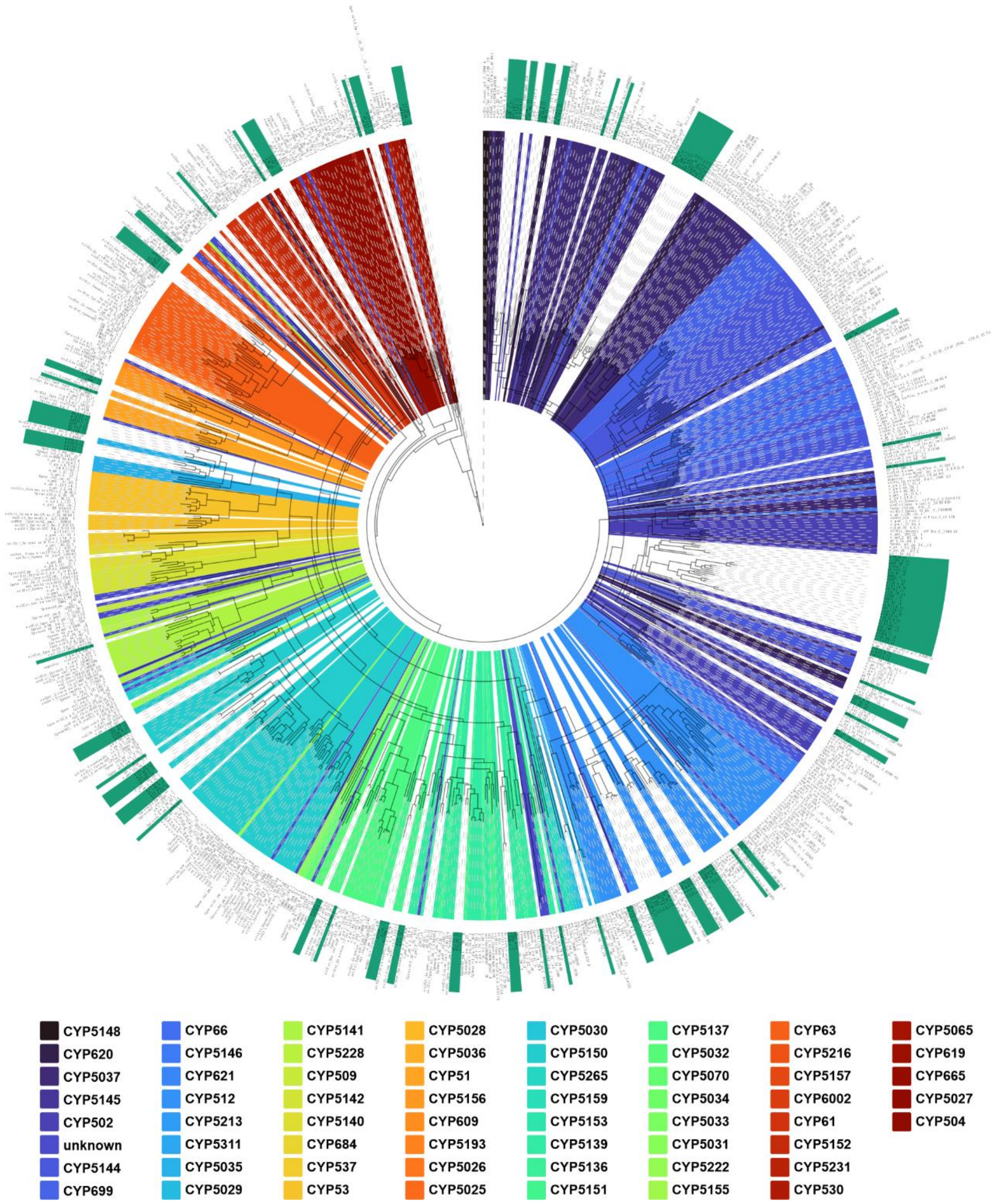

Figure S13. **P450s Cluster analysis of the strain NWAFU-1 and other Basidiomycete.**

The sequences of the branch with color background and the corresponding sequence name without background are from Fungal Cytochrome P450 Database (http://passport.riceblast.snu.ac.kr/?t=P450). The background color of the branch corresponds to the category to which it belongs. The sequences with dark green background and corresponding branch without background are P450 sequences from the strain of NWAFU-1. Multiple sequence pairs are implemented with mafft V7 .505 (https://mafft.cbrc.jp/alignment/software/) with parameters --maxiterate 1000 --localpair. The evolutionary tree was constructed by IQtreeV2.2.3 (16) with the parameters -m MFP -bb 1000 -alrt 1000 -abayes -nt AUTO.

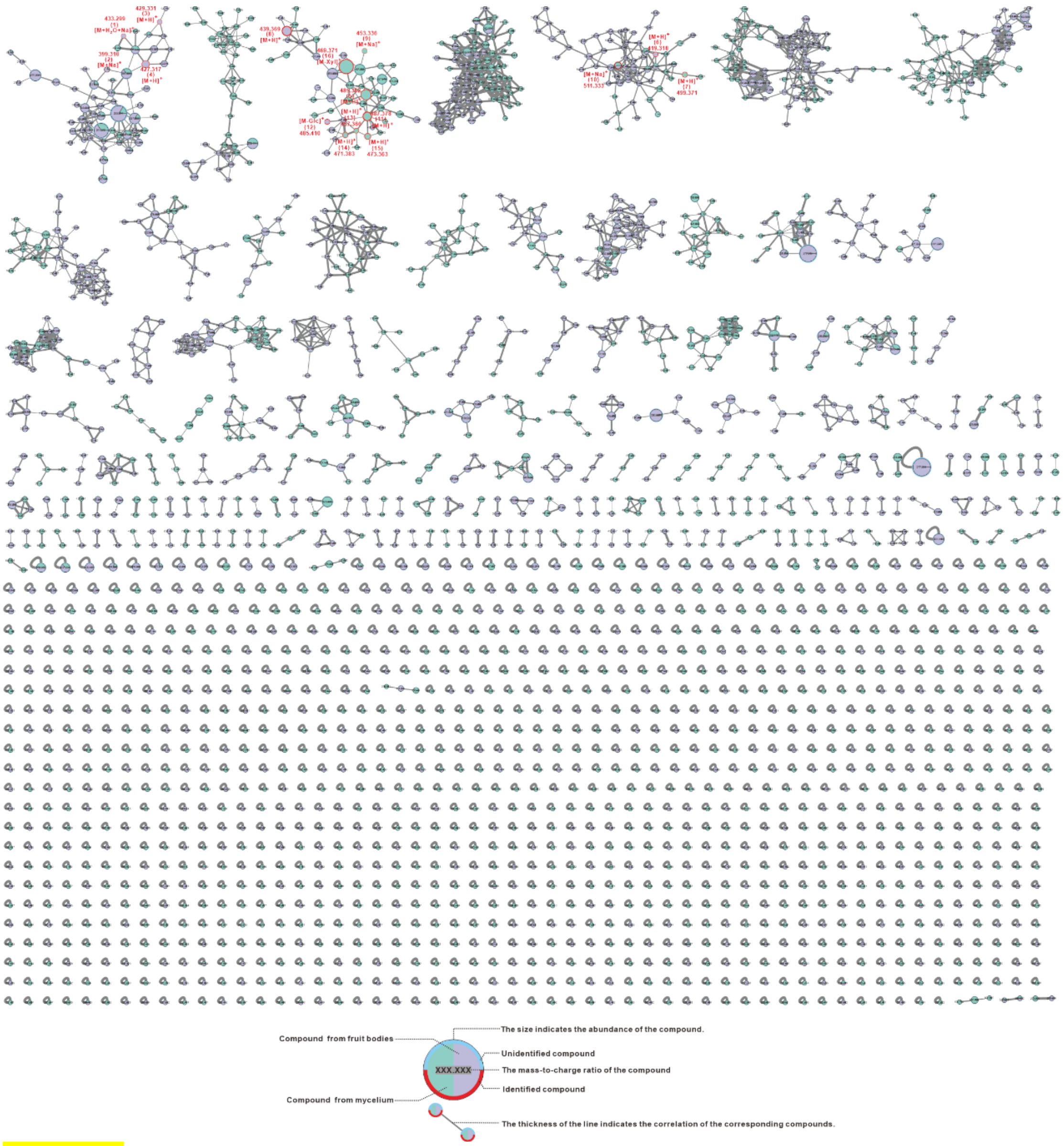

Figure S14. **Molecular network analysis of metabolites from the mycelium and fruiting bodies of the strain NWAFU-1.**

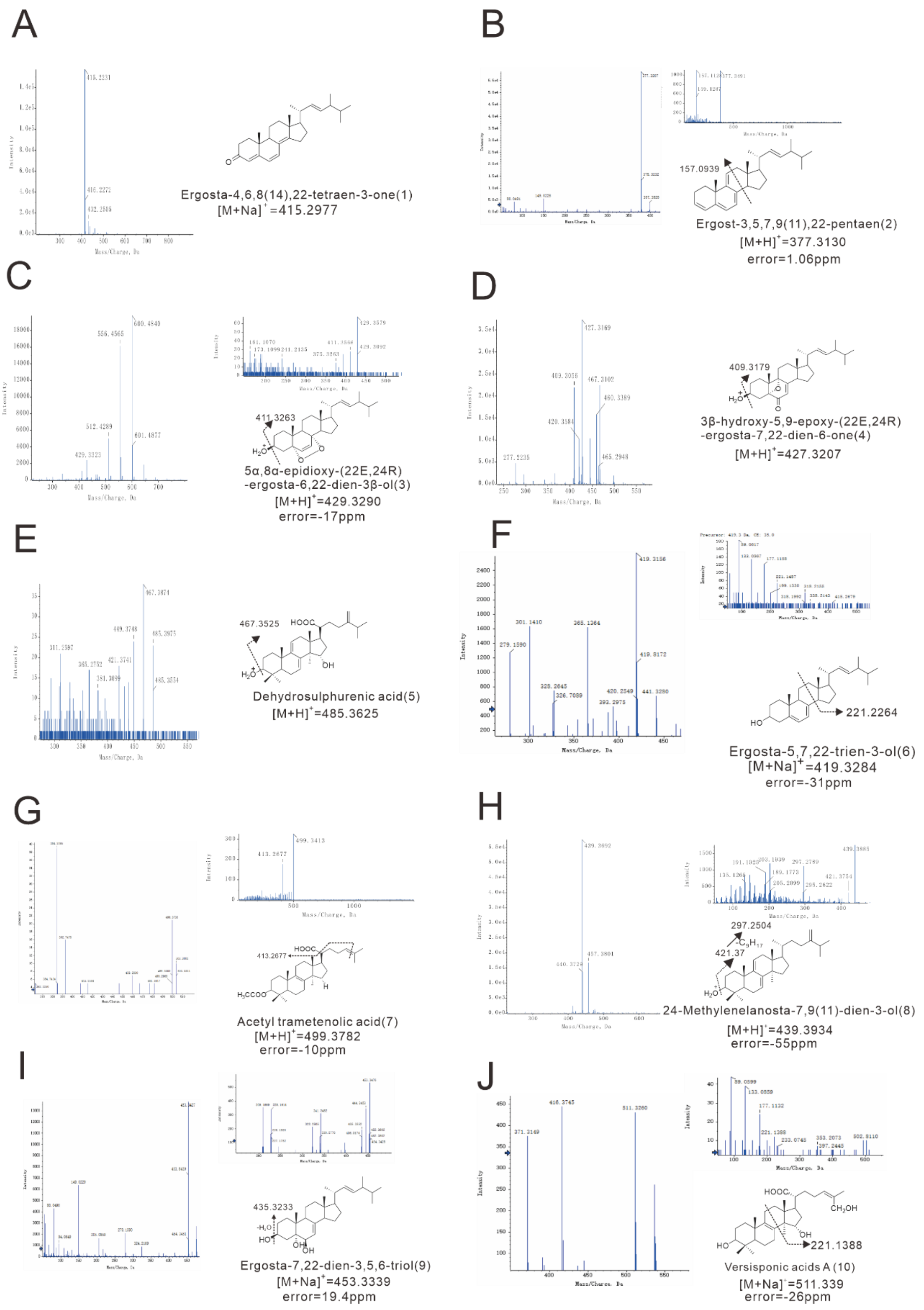
A
Ergosta-4,6,8(14),22-tetraen-3-one(1)
[M+Na]+=415.2977
B
157.0939
Ergost-3,5,7,9(11),22-pentaen(2)
[M+H]+=377.3130
error=1.06ppm
C
411.3263
5α,8α-epidioxy-(22E,24R)
-ergosta-6,22-dien-3β-ol(3)
[M+H]+=429.3290
error=-17ppm
D
409.3179
3β-hydroxy-5,9-epoxy-(22E,24R)
-ergosta-7,22-dien-6-one(4)
[M+H]+=427.3207
E
467.3525
Dehydrosulphurenic acid(5)
[M+H]+=485.3625
F
221.2264
Ergosta-5,7,22-trien-3-ol(6)
[M+Na]+=419.3284
error=-31ppm
G
413.2677
Acetyl trametenolic acid(7)
[M+H]+=499.3782
error=-10ppm
H
297.2504
421.37
24-Methylenelanosta-7,9(11)-dien-3-ol(8)
[M+H]+=439.3934
error=-55ppm
I
435.3233
Ergosta-7,22-dien-3,5,6-triol(9)
[M+Na]+=453.3339
error=19.4ppm
J
221.1388
Versisponic acids A (10)
[M+Na]+=511.339
error=-26ppm

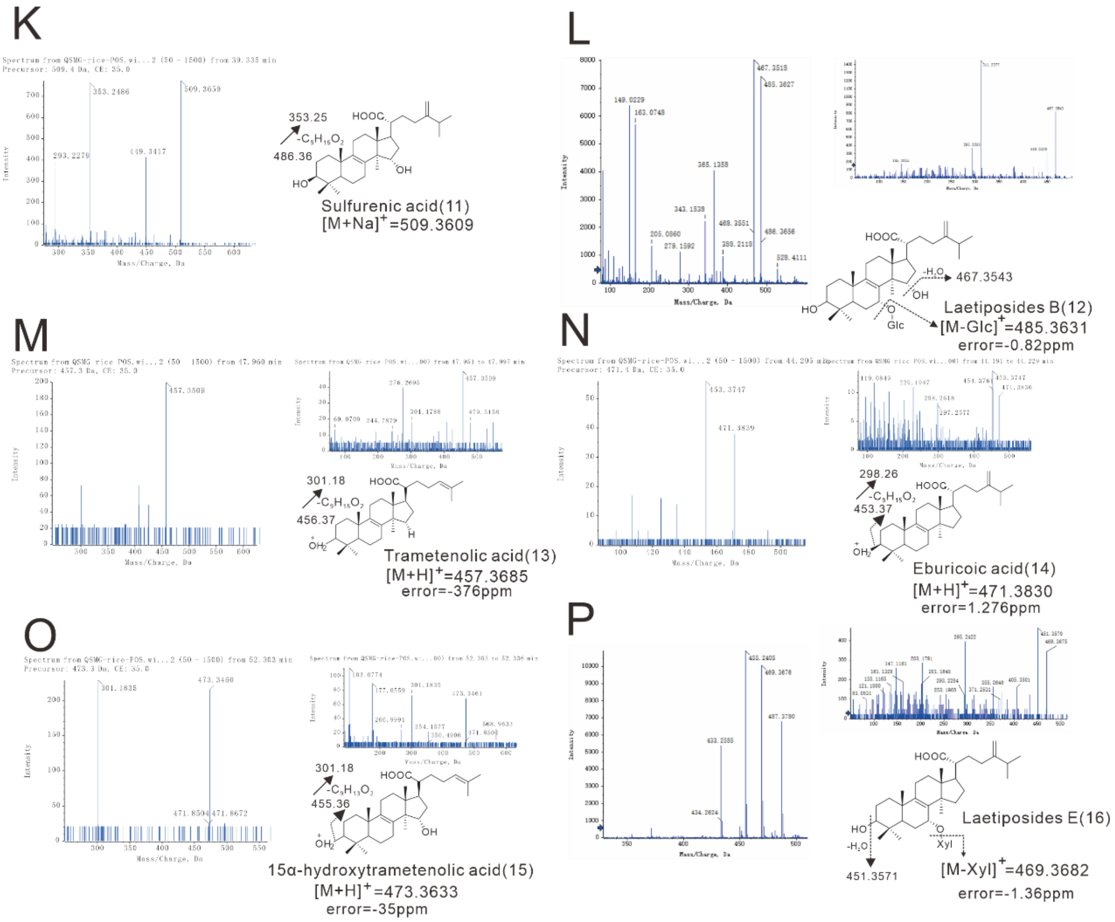

Figure S15. **The LC-ESI-HRMS and LC-ESI-HRMS/MS spectrums of isolates from the strain NWAFU-1.**

Mass spectral data were acquired in positive ion mode. Figures A-P correspond one-to-one with compounds 1-16.

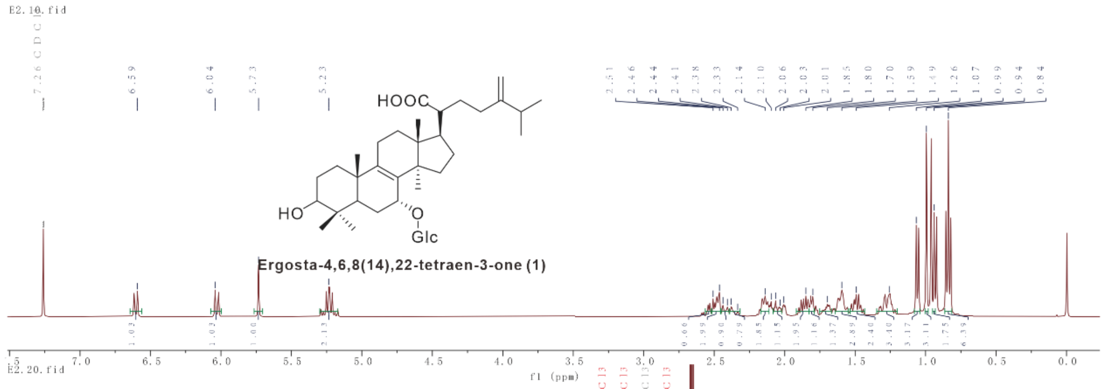
E2.10.fid
HOOC
HO
O
Glc
Ergosta-4,6,8(14),22-tetraen-3-one (1)
f1 (ppm)

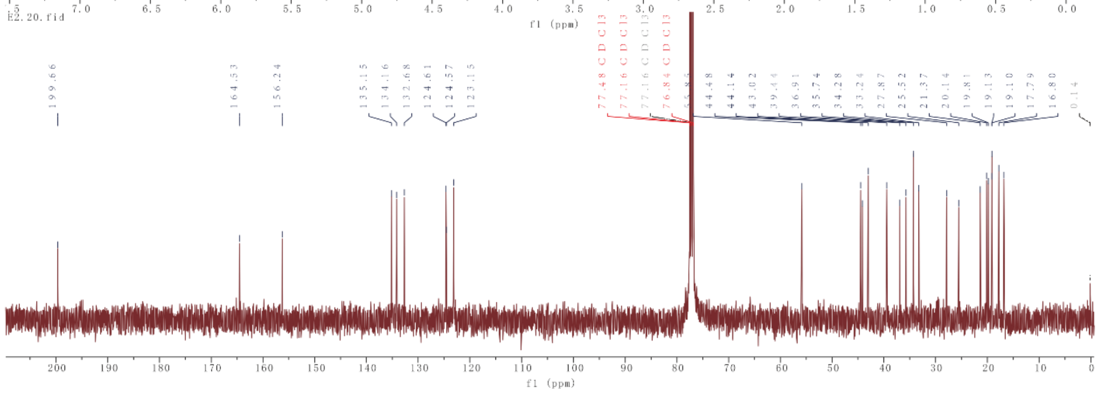
E2.20.fid
f1 (ppm)

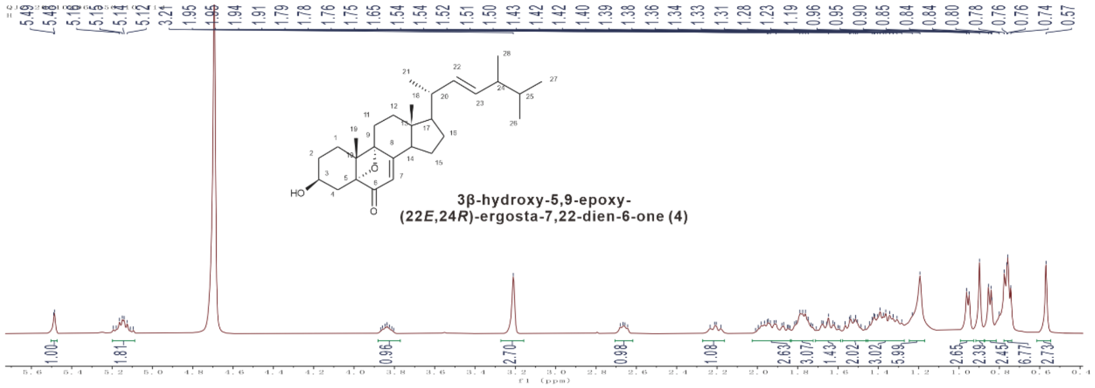
HO
O
3β-hydroxy-5,9-epoxy-
(22*E*,24*R*)-ergosta-7,22-dien-6-one (4)
f1 (ppm)

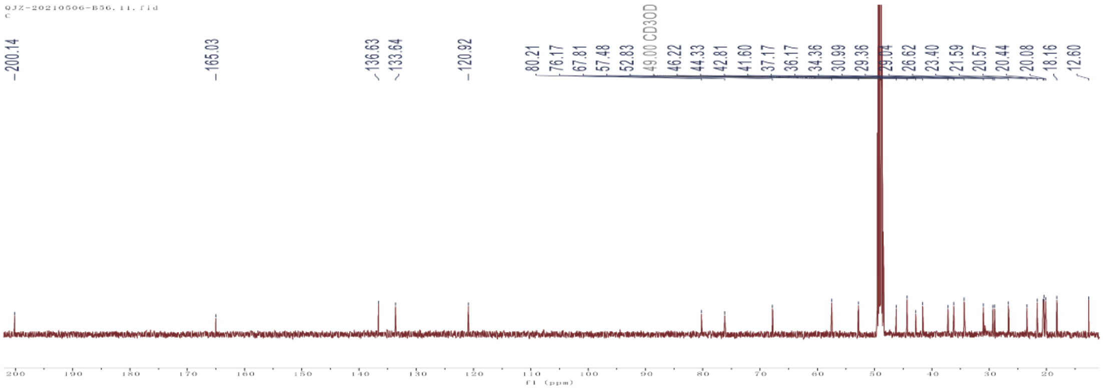
QJZ-20210506-B56.11.fid
CD3OD
f1 (ppm)

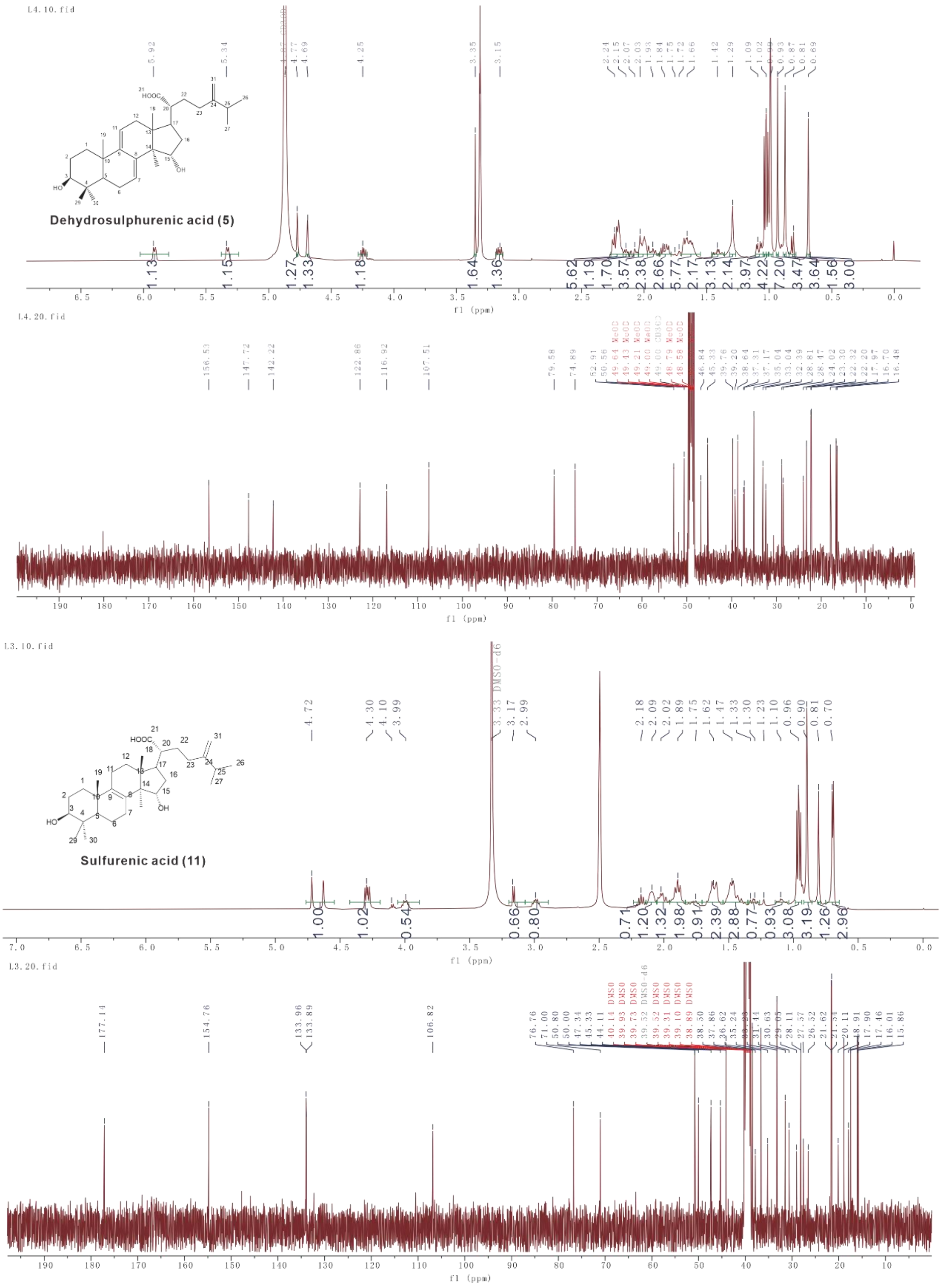

Figure S16. The NMR spectrums of compounds (1, 4, 5, and 11) from the strain NWAFU-1.

400 MHz for ^{1}H-NMR and 100 MHz for ^{13}C-NMR.

A

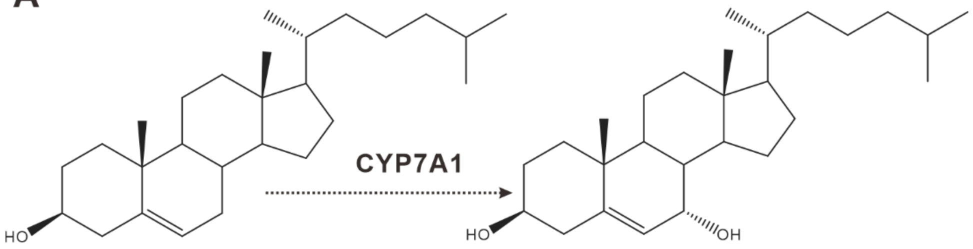

Sequences producing significant alignments:	Score (bits)	E Value
g668.t1	72	3e-013
g13485.t2	64	9e-011
g13485.t1	64	9e-011
g6896.t1	64	9e-011

B

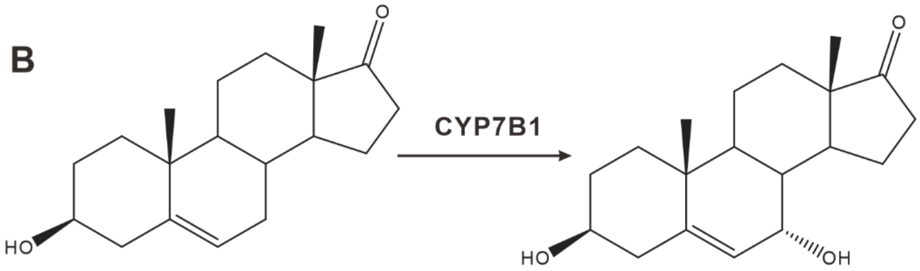

Sequences producing significant alignments:	Score (bits)	E Value
g668.t1	85	5e-017
g8402.t1	84	8e-017
g13251.t1	84	1e-016
g5483.t3	80	1e-015
g5483.t2	80	1e-015
g5024.t1	77	1e-014
g5483.t1	77	2e-014
g5018.t1	77	2e-014
g5477.t1	76	2e-014
g8113.t1	74	1e-013
g2540.t1	72	3e-013
g3955.t1	71	9e-013

Figure S17. Genome scanning of NWAFU strains was performed with C7 hydroxylases derived from *Homo sapiens* (A) and *Rattus norvegicus* (B).

The genome scanning was realized by BioEdit V7.2.5 package.

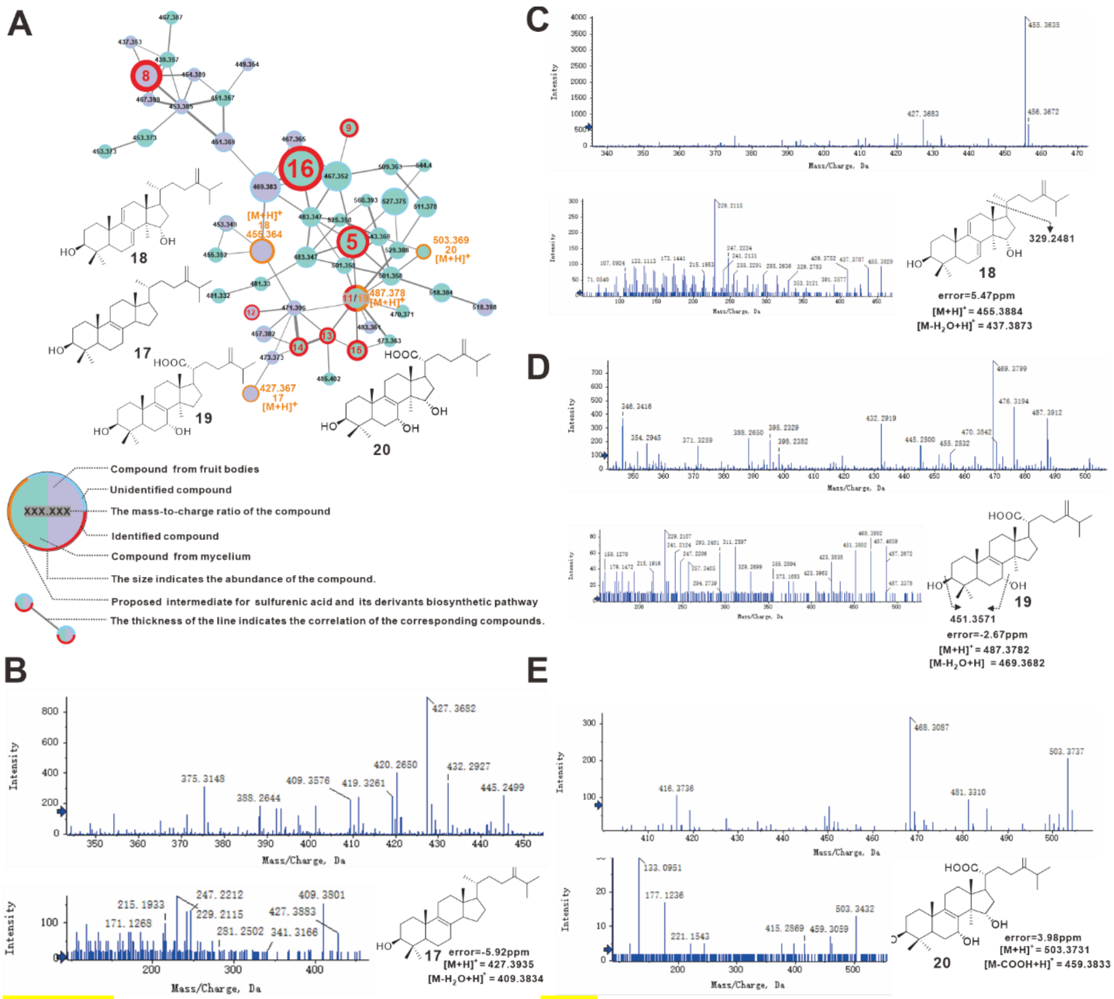

Figure S18. Identification of putative intermediates (**17-20**) by molecular network (A) and HR-MS(B-E).

Reference

1. de Figueiredo FL, de Oliveira ACP, Terrasan CRF, Gonçalves TA, Gerhardt JA, Tomazetto G, Persinoti GF, Rubio MV, Peña JAT, Araújo MF, de Carvalho Silvello MA, Franco TT, Rabelo SC, Goldbeck R, Squina FM, Damasio A. 2021. Multi-omics analysis provides insights into lignocellulosic biomass degradation by *Laetiporus sulphureus* ATCC 52600. Biotechnology for Biofuels 14:96.
2. Agger S, Lopez-Gallego F, Schmidt-Dannert C. 2009. Diversity of sesquiterpene synthases in the basidiomycete Coprinus cinereus. Mol Microbiol 72:1181-1195.
3. Quin MB, Flynn CM, Wawrzyn GT, Choudhary S, Schmidt-Dannert C. 2013. Mushroom Hunting by Using Bioinformatics: Application of a Predictive Framework Facilitates the Selective Identification of Sesquiterpene Synthases in Basidiomycota. ChemBioChem 14:2480-2491.
4. Zhang C, Chen X, Orban A, Shukal S, Birk F, Too H-P, Rühl M. 2020. *Agrocybe aegerita* Serves As a Gateway for Identifying Sesquiterpene Biosynthetic Enzymes in Higher Fungi. ACS Chemical Biology 15:1268-1277.
5. Nagamine S, Liu C, Nishishita J, Kozaki T, Sogahata K, Sato Y, Minami A, Ozaki T, Schmidt-Dannert C, Maruyama J-i, Oikawa H, Master ER. 2019. Ascomycete *Aspergillus oryzae* Is an Efficient Expression Host for Production of Basidiomycete Terpenes by Using Genomic DNA Sequences. Applied and Environmental Microbiology 85:e00409-19.
6. Xu Guang-hua S-jC, Young-hee Kim, In-ja ryoo, Soon-ja Seok, Jong-seog Ahn, andIck-dong Yoo. 2010. Secondary Metabolites of *Volvariella bombycina* and Their Inhibitory effectson Melanogenesis. Jounrnal of Microbiology and Biotechnology 20:78-81.
7. Yu FX, Li Z, Chen Y, Yang YH, Li GH, Zhao PJ. 2017. Four new steroids from the endophytic fungus *Chaetomium* sp. M453 derived of Chinese herbal medicine Huperzia serrata. Fitoterapia 117:41-46.
8. Chepkirui C, Matasyoh JC, Decock C, Stadler M. 2017. Two cytotoxic triterpenes from cultures of a Kenyan *Laetiporus sp*. (Basidiomycota). Phytochemistry Letters 20:106-110.
9. Yoshikawa K, Matsumoto K, Mine C, Bando S, Arihara S. 2000. Five Lanostane Triterpenoids and Three Saponins from the Fruit Body of *Laetiporus versisporus*. Chemical & Pharmaceutical Bulletin 48:1418-1421.
10. Chen S, Xu J, Liu C, Zhu Y, Nelson DR, Zhou S, Li C, Wang L, Guo X, Sun Y, Luo H, Li Y, Song J, Henrissat B, Levasseur A, Qian J, Li J, Luo X, Shi L, He L, Xiang L, Xu X, Niu Y, Li Q, Han MV, Yan H, Zhang J, Chen H, Lv A, Wang Z, Liu M, Schwartz DC, Sun C. 2012. Genome sequence of the model medicinal mushroom *Ganoderma lucidum*. Nature Communications 3:913.
11. Kües U, Nelson DR, Liu C, Yu G-J, Zhang J, Li J, Wang X-C, Sun H. 2015. Genome analysis of medicinal *Ganoderma spp*. with plant-pathogenic and saprotrophic life-styles. Phytochemistry 114:18-37.
12. Ohm RA, de Jong JF, Lugones LG, Aerts A, Kothe E, Stajich JE, de Vries RP, Record E, Levasseur A, Baker SE, Bartholomew KA, Coutinho PM, Erdmann S, Fowler TJ, Gathman AC, Lombard V, Henrissat B, Knabe N, Kües U, Lilly WW, Lindquist E, Lucas S, Magnuson JK, Piumi F, Raudaskoski M, Salamov A, Schmutz J, Schwarze FWMR, vanKuyk PA, Horton JS, Grigoriev IV, Wösten HAB. 2010. Genome sequence of the model mushroom *Schizophyllum commune*. Nature Biotechnology 28:957-963.
13. Chen C-L, Li W-C, Chuang Y-C, Liu H-C, Huang C-H, Lo K-Y, Chen C-Y, Chang F-M, Chang G-A, Lin Y-L, Yang W-D, Su C-H, Yeh T-M, Wang T-F, Farrer RA. 2022. Sexual Crossing, Chromosome-Level Genome Sequences, and Comparative Genomic Analyses for the Medicinal Mushroom *Taiwanofungus Camphoratus* (Syn. *Antrodia Cinnamomea*, *Antrodia Camphorata*). Microbiology Spectrum 10:e02032-21.
14. Gilchrist CLM, Chooi YH. 2021. Clinker & clustermap.js: Automatic generation of gene cluster comparison figures. Bioinformatics doi:10.1093/bioinformatics/btab007.
15. Navarro-Munoz JC, Selem-Mojica N, Mullowney MW, Kautsar SA, Tryon JH, Parkinson EI, De Los Santos ELC, Yeong M, Cruz-Morales P, Abubucker S, Roeters A, Lokhorst W, Fernandez-Guerra A, Cappelini LTD, Goering AW, Thomson RJ, Metcalf WW, Kelleher NL, Barona-Gomez F, Medema MH. 2020. A computational framework to explore large-scale biosynthetic diversity. Nature Chemical Biology 16:60-68.
16. Minh BQ, Schmidt HA, Chernomor O, Schrempf D, Woodhams MD, von Haeseler A, Lanfear R. 2020. IQ-TREE 2: New Models and Efficient Methods for Phylogenetic Inference in the Genomic Era. Molecular Biology and Evolution 37:1530-1534.
